# Advances in the greener synthesis of chromopyrimidine derivatives by a multicomponent tandem oxidation process

**DOI:** 10.1038/s41598-023-46004-3

**Published:** 2023-11-04

**Authors:** Pouya Ghamari Kargar, Ghodsieh Bagherzade

**Affiliations:** Department of Chemistry, Faculty of Sciences, University of Birjand, Birjand, 97175-615 Iran

**Keywords:** Catalysis, Green chemistry, Organic chemistry, Chemical synthesis

## Abstract

A hydrophilic cobalt/copper heterogeneous bimetallic catalyst named mTEG-CS-Co/Cu-Schiff-base/IL was successfully synthesized from chitosan polysaccharide. The new catalyst was investigated and confirmed using various techniques including FT-IR, FE-SEM, EDX-EDS, XRD, TEM, TGA, AFM, NMR and ICP. The catalyst exhibited powerful catalyst activity for the tandem one pot oxidative chromopyrimidine reaction from benzyl alcohols under mild conditions, utilizing air as a clean source in a green protocol. The catalyst was compatible with a wide range of benzyl alcohols, and aldehydes formed in situ, and bis-aldehydes synthesized were condensed with urea/4‑hydroxycumarin to provide favorable products in good yields for all derivatives (14 new derivatives). The presence of tri-ethylene glycol and imidazolium moieties with hydrophilic properties on the mTEG-CS-Co/Cu-Schiff-base/IL nanohybrid provides dispersion of the nanohybrid particles in water, leading to higher catalytic performance. Furthermore, the reaction exhibited several other notable features, including low catalyst loading, the ability to be recycled for up to 6 stages, high atom economy, a simple work procedure, short reaction time, utilization of an environmentally friendly nanohybrid, and the replacement of volatile and organic solvents with water solvent.

## Introduction

Sustainable and green chemistry aims to develop environmentally friendly methods for synthesizing biologically significant compounds, such as heterocyclic compounds^1, 2^. The development of cost-effective and reliable methodologies is crucial for the sustainability of green chemistry, the use of benign solvents, and the expansion of less toxic and promising catalysts^3, 4^. Heterocyclic compounds are molecules with cyclic structures that contain at least one atom, such as S, O, or N in addition to carbon^5, 6^. These compounds have diverse pharmacological properties and are used clinically. Pharmacologically, they inhibit simple voltage-gated receptors and modulate highly complex signaling cascades in cells^7, 8^. Among the heterocycles containing nitrogen, oxygen, and sulfur, chromenes and pyrimidines are important classes of heterocyclic compounds. Many drugs and bioactive natural products contain pyrimidine and chromene as privileged moieties^9–11^.

**Figure 1:**
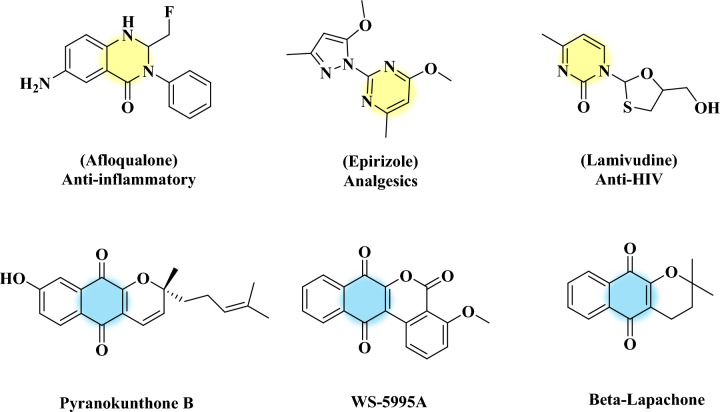
Structures of some biologically active pyrimidine and chromene molecules.

The pyrimidine and chromene rings are commonly found in nature among heterocyclic compounds^12, 13^. Pyrimidine derivatives, such as thymine, cytosine, and uracil, are essential components of nucleic acids^14^. Certain pyrimidine compounds, including veronal and barbituric acid, have hypnotic properties^15^. Moreover, pyrrolo [3,2-d] pyrimidine derivatives act as antagonists to adenosine receptor subtypes and enzyme inhibitors. The structural similarity of pyrimidine molecules to flavins contributes to their medicinal significance^16^. Pyrimidine fused quinoline derivatives have a wide range of medicinal properties, including antibacterial, antifungal, anticancer, analgesic, antimalarial, antitumor, anti-inflammatory, antiviral, and antioxidant effects^17–19^. Also, chromene derivatives have diverse pharmacological properties, such as anticancer^20^, antimicrobials^21^, antivirals^22^, anticoagulants^23^, and anti-inflammatory activities^24^. Therefore, it is crucial for the pharmaceutical industry to develop synthetic methods for chromene derivatives. Figure 1 illustrates the structure of some biologically active molecules containing chromene and pyrimidine blocks^25^. Over the years, a variety of synthetic methods have been developed for pyrimidine or chromene analogs. Among these methods, catalyzed reactions by transition metals are the most attractive approaches for synthesizing chromene and pyrimidine derivatives^26, 27^. Since pyrimidines and chromenes have numerous applications, developing new and efficient routes for synthesizing pyrimidine-fused chromene derivatives has remained a crucial area of research. The fusion of pyrimidines and chromenes enhances their biological properties, and chromopyrimidines are used in a range of drugs, including antiplatelets and antithyroid drugs^28, 29^.

Multiplex-modified catalysts have been utilized for the synthesis of chromopyrimidines and their derivatives. However, several reports have highlighted issues such as long reaction times, labor-intensive processes, the use of non-recyclable catalysts, and low product yields^30–32^. Also, chromopyrimidines can be synthesized from alcohols through multicomponent TOP (tandem oxidation process) reactions^33, 34^. Tandem reactions have attracted considerable attention due to their ability to combine several reactions in a vessel, reducing waste production, and eliminating intermediate separations. Among organic reactions, alcohols are considered environmentally safe chemicals due to their wide accessibility, low cost, availability from renewable biomass sources, low toxicity, and ease of use, storage, transport, and dissolution^35, 36^. The concept of TOP was pioneered by Robert Ireland during his attempts to synthesize poly-ether ionosphere antibiotics^37^. However, the challenge of separating large quantities of aldehydes produced during the process was addressed by oxidizing alcohols with the Swern reagent and introducing the Wittig reagent to the in situ generated aldehydes^38^. Since its discovery, TOP has been employed in the synthesis of a wide range of useful materials from alcohols. In recent years, various oxidizing and nucleophilic compounds have been employed in TOP reactions^39, 40^. Further, TOP has been extended to include multicomponent reactions (MCRs), enabling the synthesis of diverse valuable compounds^41–43^. These processes provide a high atom economy, reducing the need for time-consuming, expensive, waste-generating operations, and purification processes in chemical synthesis^44^. Most recently, multifunctional catalysts have been developed by combining different types of active sites on catalysts for use in tandem catalysis processes. A new area of research focuses on the application of multifunctional catalytic systems in multicomponent TOPs starting from alcohols^45, 46^.

Material or sustainable chemistry focuses on the developiment of safe materials with catalytic properties^47–49^. Catalysts play a crucial role in lowering the activation energy and increasing the rate of chemical reactions^50, 51^. Green chemistry emphasizes the reusability and sustainability of catalytic materials, highlighting the need for environmentally friendly, sustainable, and economically effective catalytic systems. Carbon, being abundantly available in nature, has garnered significant attention in research worldwide for its potential as a green catalyst. When designing a catalyst and selecting the appropriate substrate, all aspects of the catalyst's design must be considered, including non-toxicity, biodegradability, environmental friendliness, renewability, abundance, as well as the surface area and morphological attributes of the material^52–54^.

In this context, chitosan (CS) is recognized as an ideal material with remarkable properties. It possesses active functional groups (hydroxyl: -OH, amino: -NH_2_), exhibits non-toxic, high oxidation stability, a large surface area, exceptional physical strength, excellent electron conductivity, and good chemical integrity^55, 56^. However, CS does have certain drawbacks, including solubility in dilute acids, easy aggregation, and poor mechanical strength. Therefore, improving the development of mechanical properties, surface area, and chemical stability of CS is of great importance^57^. Various conventional techniques have been employed to enhance the properties of CS, such as grafting, cross-linking, and composting. Among them, cross-linking reactions of CS are one of the most suitable chemical modification techniques that can enhance the chemical stability, absorption ability, mechanical strength, solubility in acidic environment, and hydrophobicity of CS^58, 59^. So far, various applications of chitosan have been reported, including the removal of radioactive elements, wastewater treatment, catalyst, medicine, fabric production, food, cosmetics, agriculture, biotechnology, nutrition and photography^60–65^. Recently, chitosan-based catalysts in water environments with fast recycling capabilities have gained attention from both environmental and economic perspectives^66^. Water, due to its green credentials such as high specific heat capacity, high polarity, a network of hydrogen bonds, high surface tension, high cohesive energy, low cost, and easy accessibility, has emerged as a potential solvent for organic reactions in recent years^67, 68^.

Eco-friendliness in chemical processes often refers to the use of sustainable and environmentally benign methods. Ionic liquids, known as molten salts, are a fascinating class of chemical compounds consisting of organic cations and organic or inorganic anions. However, they possess distinct properties that set them apart from traditional ionic salts^69^. The unique characteristics of ionic liquids heavily rely on the choice of cations and anions. By selecting different combinations of cations and anions, a wide range of applications for ionic liquids can be achieved. When it comes to Ionic liquids (ILs), they have gained attention as potential alternatives to traditional organic solvents due to their unique properties, such as low volatility, high thermal stability, tunable physicochemical properties, and low vapor pressure^70, 71^. For this reason, they have been used as green solvents in most organic reactions, including hydrogenation, oxidation, coupling reactions, Diels–alder reaction, etc^72^. However, their high cost and high viscosity have limited their application in organic reactions, as only a thin layer known as the diffusion layer effectively participates in the reaction process. To overcome this limitation, nanocatalysts containing ionic groups have been introduced to address concerns regarding immobilization and recoverability associated with ILs^73, 74^. This approach enhances the "ionophilicity" of the reagent in the reaction media, facilitating the exchange of counter ions with the leaving group of the reagents^75, 76^. Additionally, metal complexes bearing an imidazolium moiety can serve as bases and minimize metal leaching in the reaction media. Several comprehensive reviews and articles have emphasized the usefulness of catalytic metal ionic liquids in organic synthesis. These ILs have been successfully employed as recyclable nanocatalysts, offering green and efficient solutions for a wide range of organic transformations. Significant contributions have been made in various areas, including CO_2_ conversion, biodiesel formation, coupling reactions, condensation reactions, acetylation, esterification, and multi-component reactions^77^. Over time, numerous biologically significant molecules have been extensively synthesized using catalytic ILs through multi-component reactions including chromenes^78^, 1,2,3-triazoles^79^, dihydropyrano[2,3-c]coumarin^80^, 2H-indazolo[2,1-b] phthalazine-trione^81^, α-amino-phosphonates^82^, furan^83^, pyrano[2,3-d]pyrimidinone^84^, xanthenes^85^, Diels–Alder reaction^86^ and etc^87, 88^.

**Figure 2:**
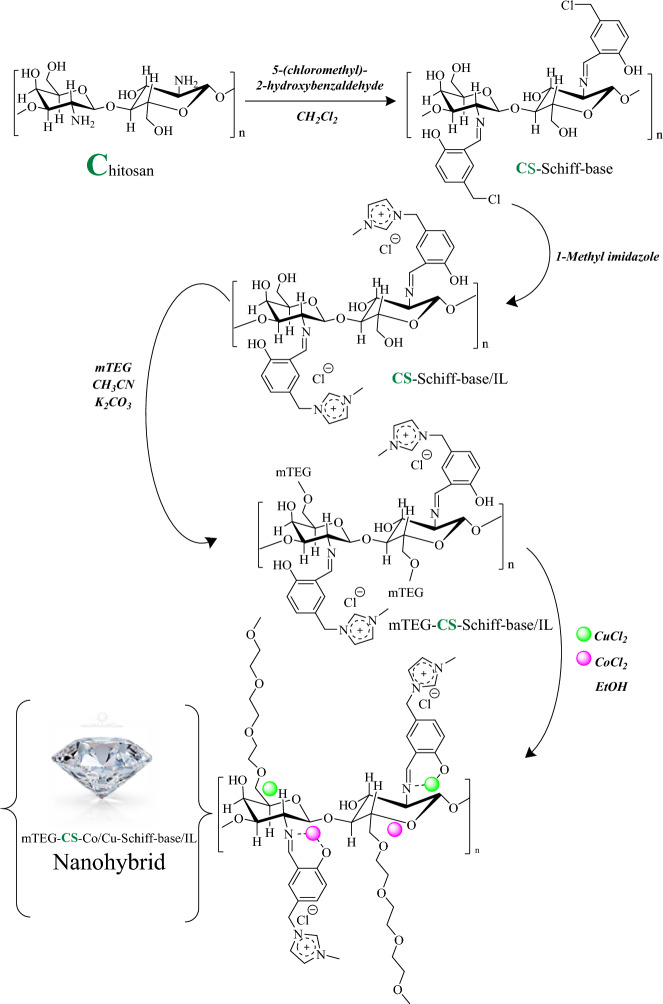
Schematic illustration of mTEG-CS-Co/Cu-Schiff-base/IL nanohybrid synthesis strategy.

Finally, given the wide range of applications of chitosan in various industries, it has been utilized as a catalyst in multicomponent reactions. Previous research has shown that the oxidation of alcohols to aldehydes is a highly selective and controllable reaction. However, there are no existing reports on the synthesis of chromopyrimidine derivatives from alcohols or the symmetrical synthesis of these compounds using bis-aldehyde derivatives. Therefore, we have developed an efficient and cost-effective one-pot tandem method for the synthesis of chromopyrimidine derivatives using readily available substrates and catalysts. In this study, we synthesized a recyclable cobalt/copper heterogeneous bimetallic nanohybrid from chitosan (Fig. 2: mTEG-CS-Co/Cu-Schiff-base/IL) that exhibited excellent performance in the tandem oxidation-condensation reaction of chromopyrimidine from alcohols. The cooperative, and synergistic effects in mTEG-CS-Co/Cu-Schiff-base/IL were investigated through various control experiments (Fig. 2).

## Experimental

All the specifications of chemicals and devices used are available in the support information file.

### Preparation of the CS-Schiff-base/IL

The synthesis of the chitosan was performed according to an already-established procedure^89^. Firstly, purified CS (1000 mg) was dissolved in 80 mL of a diluted 1% (w/w) aqueous AcOH solution at room temperature for 12 h. In another round bottom flask, 0.016 mol of 5-(chloromethyl)-2-hydroxybenzaldehyde^90–92^ was dissolved in CH_2_Cl_2_. Then, the aldehyde solution was added dropwise to the CS solution under stirring and the mixture was heated to 50 °C for 5 h. After the end of the reaction, 0.016 mol of 1-Methyl imidazole was added to the CS-Schiff-base solution, and the mixture was refluxed for 24 h. Finally, the reaction mixture was collected by centrifugation, and washed with CH_2_Cl_2_ (3 × 10 mL) to remove any unreacted imidazole, and aldehyde and dried in a vacuum oven at 40 °C for 12 h.

### Preparation of the mTEG-CS-Schiff-base/IL

In this method, the mTEG-CS-Schiff-base/IL is prepared by stirring a mixture of mTEG (tosylate mono-methoxy triethylene glycol: 20 mmol), (1 g), and K_2_CO_3_ (20 mmol) in dry acetonitrile (50 mL) at 60 °C for 24 h. The resulting solid material is separated by centrifugation, washed with water and ethanol (3 × 10 mL), and then dried in an oven at 50 °C for 24 h.

### Preparation of the mTEG-CS-Co/Cu-Schiff-base/IL nanohybrid

Finally, the mTEG-CS-Co/Cu-Schiff-base/IL nanohybrid is prepared by adding CoCl_2_ (2 mmol) and CuCl_2_ (2 mmol) to the mixture of mTEG-CS-Schiff-base/IL (1 g) in EtOH (20 mL) and stirring at 80 °C for 24 h. After the reaction, the mixture is cooled, filtered off, washed with ethanol (2 × 20 mL), and dried in an oven at 60 °C for 24 h. The amount of Co and Cu loading on the nanohybrid is measured using Atomic Absorption Spectroscopy (AAS) and Inductively Coupled Plasma (ICP) instruments to obtain certainty about the results. The results from both analyses show a small difference of 0.006 between them. The analysis shows that the amount of copper and cobalt in one gram of the catalyst is 0.60 mmol and 0.52 mmol, respectively, and the percentage of the metals is 6.8%w and 6.3%w for Cu and Co, respectively. For comparison, the monometallic copper and cobalt catalysts synthesized separately have metal loadings of 7.9% w/w for Cu and 7.4% w/w for Co in one gram of catalyst, respectively.

### General procedure for the tandem one‑pot preparation of chromenopyrimidine-2,5-dione reaction with alcohol

A mixture of alcohol (1.2 mmol), urea (1 mmol), and 4-hydroxycoumarin (1 mmol) in the presence of the nanohybrid (3 mg) was stirred in H_2_O (5 ml) at the appropriate time and temperature. The progress of the reaction was cheeked by TLC. Once the desired reaction is complete, the catalyst is removed, and the product is allowed to precipitate. Finally, the products were purified by recrystallization from EtOH (See spectral data in SI File).

### General procedure for the chromenopyrimidine-2,5-dione and thioxochromeno pyrimidin-5-one reactions with Bis-aldehyde

The synthesis of bis-aldehyde derivatives was conducted following an established procedure^93, 94^. The one-pot synthesis of Chromenopyrimidine-2,5-dione was carried out in a round-bottom flask using bis-aldehyde (1 mmol), urea (2 mmol), and 4-hydroxycoumarin (2 mmol) in the presence of mTEG-CS-Co/Cu-Schiff-base/IL nanohybrid (4 mg), under aerobic conditions in H_2_O at 50 °C. The progress of the reaction was monitored using TLC. Finally, the products were purified by recrystallization from EtOH (See spectral data in SI File).

## Result and discussion

Nanohybrid was synthesized as predestined in Fig. 2. Initially, 5-(chloromethyl)-2-hydroxybenzaldehyde is coated with chitosan to obtain CS-Schiff-base. The CS-Schiff-base/IL is then formed by reacting the chlorine groups on the Schiff base chains with methyl imidazole. To introduce a phase transfer functional group, monomethoxy triethylene glycol (mTEG) is added to the hydroxyl group of the CS-Schiff-base/IL. This addition enhances the solubility and stability of the nanohybrid. Finally, cobalt and copper nanoparticles are immobilized on mTEG-CS-Co/Cu-Schiff-base/IL by adsorption of CoCl_2_ and CuCl_2_ on the nanohybrid. The synthesized catalyst was characterized using various techniques including XRD, FE-SEM, TEM, TGA, EDX-EDS, FT-IR, AFM, and ICP.

**Figure 3:**
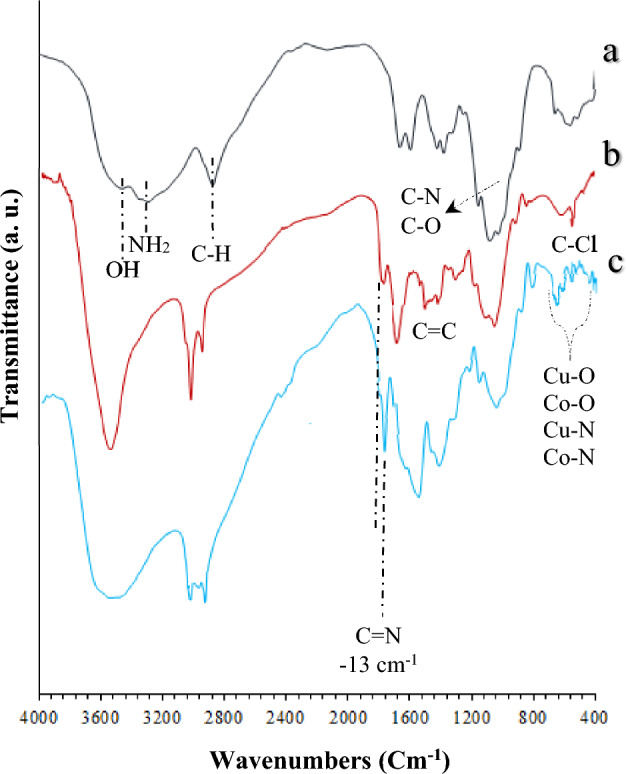
FT-IR spectra of (**a**) Chitosan, (**b**) CS-Schiff-base/IL and (**c**) mTEG-CS-Co/Cu-Schiff-base/IL nanohybrid.

FT-IR spectra are one of the best instruments to provide adequate data to clarify the bonding of the synthesized compounds. Figure 3 shows the FT-IR spectra of pure chitosan, CS-Schiff-base/IL and mTEG-CS-Co/Cu-Schiff-base/IL nanohybrid. In Fig. 3a (CS), a strong peak is observed in the FT-IR spectra of chitosan in the region range of 3000 and 3600 cm^−1^, corresponding to the stretching vibrations of amine and hydroxyl groups. The bands observed at 2876, 1643, 1074, and 1022 cm^−1^ can be attributed to the C-H aliphatic, N–H bending, C-N, and C-O stretching, respectively. In the spectrum of CS-Schiff-base/IL (Fig. 3b), the absorption bands around 3101, 1690, 1500–1598, and 753 cm^−1^ are assigned to the stretching vibration of C-H aromatic ring, C = N Schiff base/imidazole, C = C aromatic/imidazole ring, and C–Cl stretch bonds. Comparing the mTEG-CS-Co/Cu-Schiff-base/IL spectrum with the CS-Schiff-base/IL spectrum, it is evident that most of the characteristic peaks are stronger in the former spectrum. This enhancement is attributed to the addition of the mTEG chain and Co/Cu metal ions. However, mTEG-CS-Co/Cu-Schiff-base/IL spectrum indicates the formation of nanohybrid groups (Fig. 3c). Notably, compared to CS and CS-Schiff-base/IL, the mTEG-CS-Co/Cu-Schiff-base/IL spectrum exhibits multiple new bands at 463, 461, 542, and 625 cm^−1^, corresponding to Cu–N, Co–N, Cu–O, and Co–O groups, respectively. These bands indicate the successful stabilization of metallic groups^95^. Furthermore, the FT-IR spectrum of the mTEG-CS-Co/Cu-Schiff-base/IL nanohybrid demonstrates a shift (13 cm^−1^) in the stretching vibration of C = N groups to lower wavenumbers. This shift confirms the coordination of Co(II) and Cu(II) metal ions with the nitrogen and oxygen atoms of the Schiff base.

**Figure 4:**
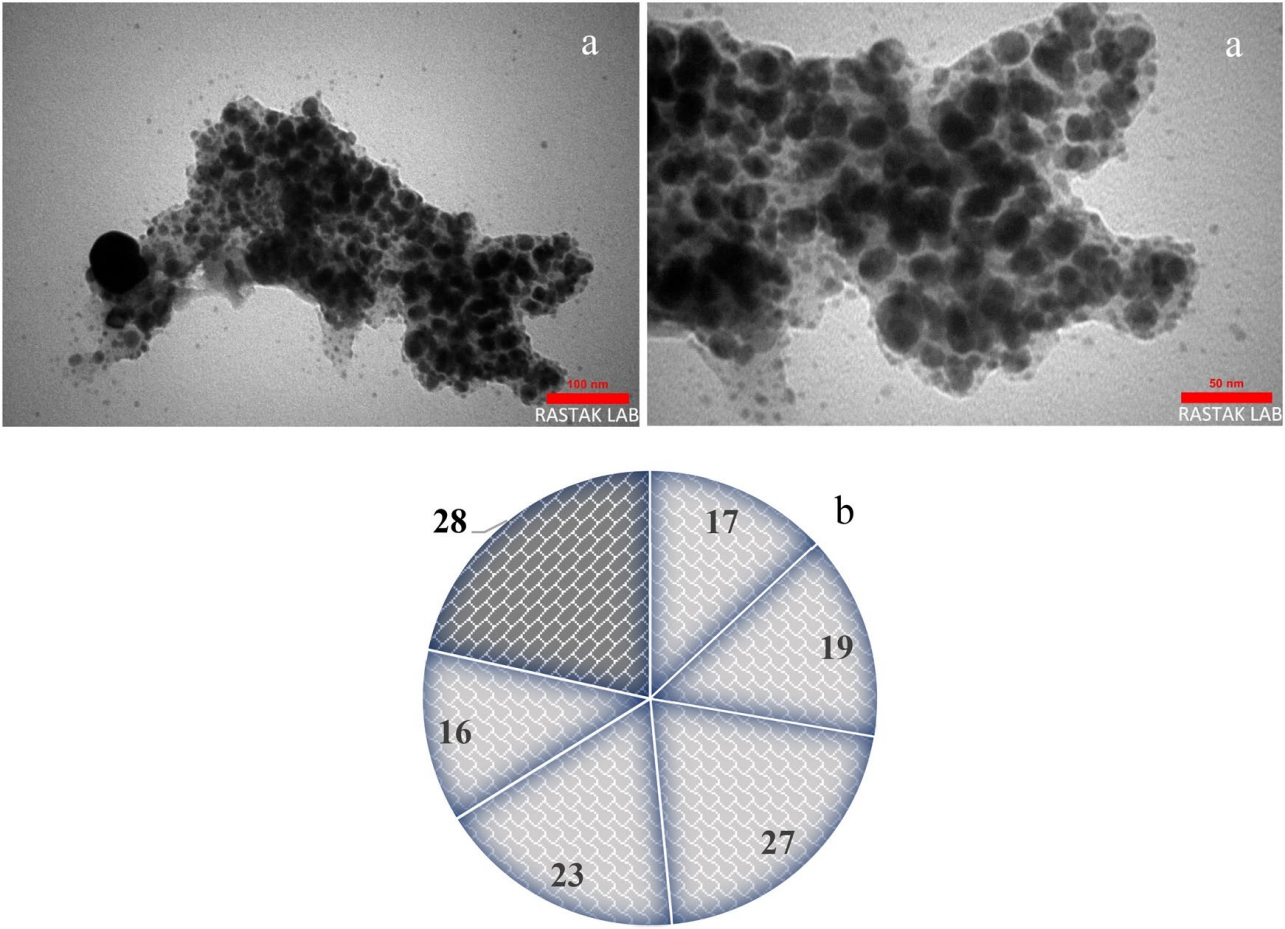
TEM image (**a**) and Particle size distribution histogram (**b**) of mTEG-CS-Co/Cu-Schiff-base/IL nanohybrid.

Figure 4 shows that the nanosized mTEG-CS-Co/Cu-Schiff-base/IL nanohybrid exhibits no evidence of aggregation, and all particles are nearly spherical in shape. The largest particle size observed in the sample is 28 nm, which is found in the embedded mTEG-CS-Co/Cu-Schiff-base/IL nanohybrid.

**Figure 5:**
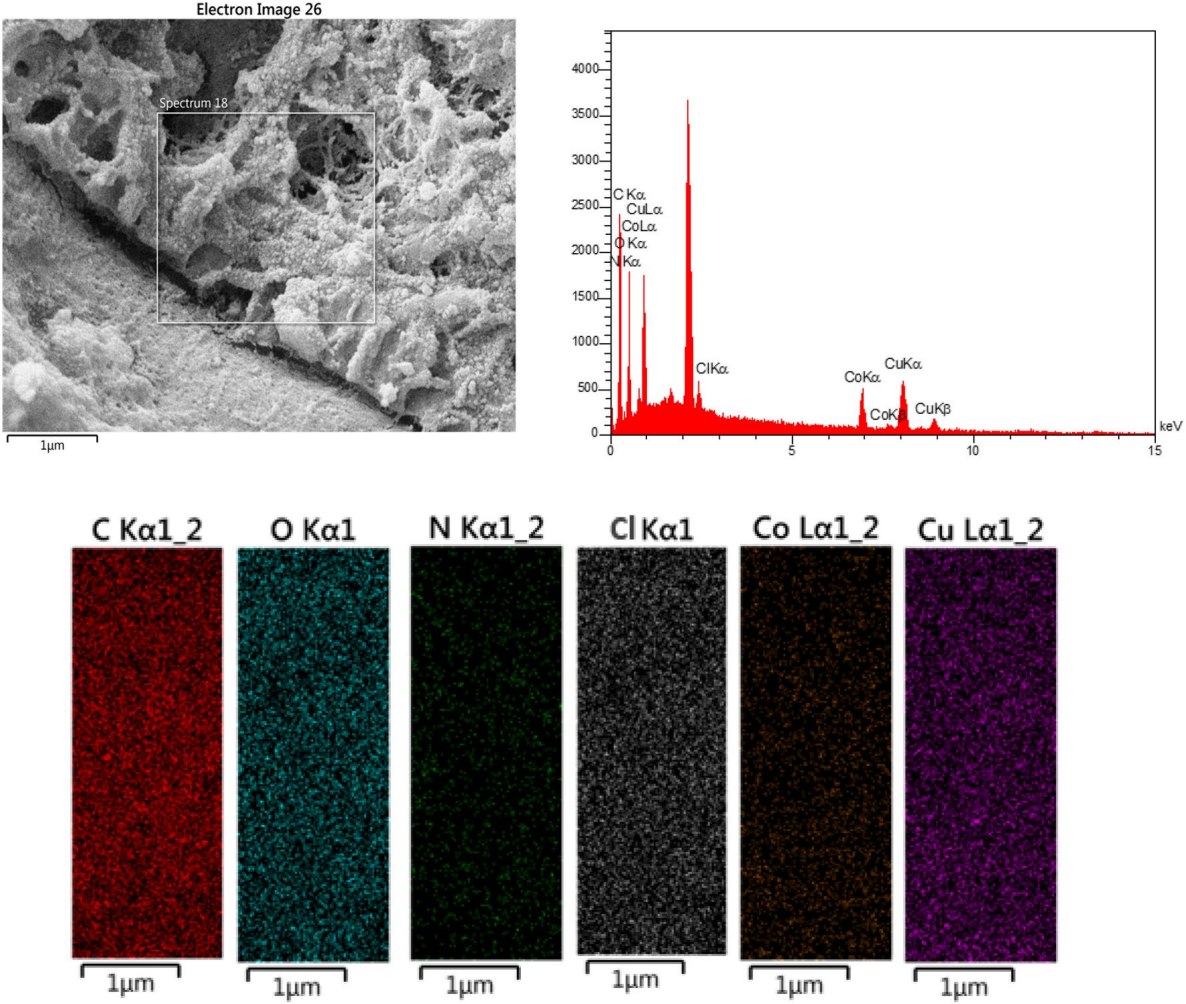
EDX-EDS spectra and elemental mapping of mTEG-CS-Co/Cu-Schiff-base/IL nanohybrid.

Based on the Energy Dispersive X-Ray (EDX) analysis depicted in Fig. 5, it is evident that the nanohybrid can be successfully functionalized to serve as a catalyst. The EDX spectrum shows the presence of various elements in the nanohybrid, that the weight percentages of these elements in the nanohybrid are as follows: 51.2% for C, 17.5% for O, 5.2% for N, 12.4% for Cu, 11.5% for Co, and 2.1% for Cl. Furthermore, the EDS spectrum of the nanohybrid confirms its full chemical complexity, indicating that the nanocomposite comprises a combination of these elements in a chemically integrated manner.

**Figure 6:**
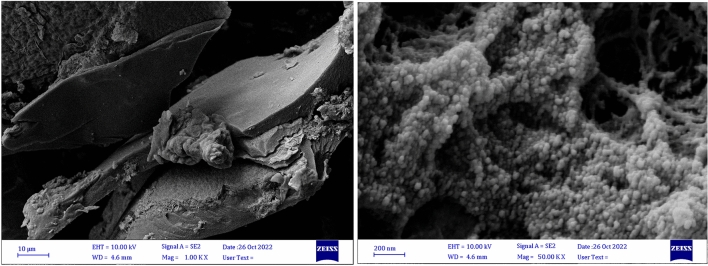
FE-SEM images of mTEG-CS-Co/Cu-Schiff-base/IL nanohybrid.

The surface morphologies of the nanohybrid were visualized directly using FE-SEM (Fig. 6). As shown in Fig. 6, the roughness illustrated on the surface of the nanohybrid indicates chemical modification of the chitosan surface.

**Figure 7:**
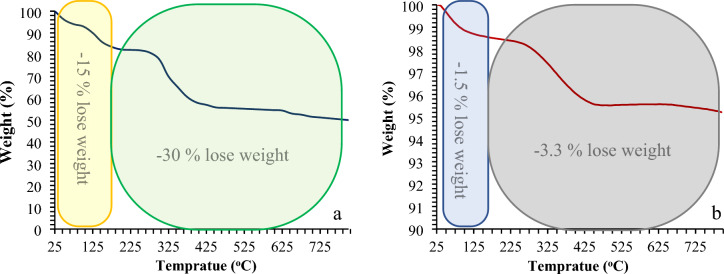
TGA weight-loss curve for the mTEG-CS- Schiff-base/IL (**a**) and mTEG-CS-Co/Cu-Schiff-base/IL (**b**) nanohybrid.

In Fig. 7, the TGA data from mTEG-CS-Schiff-base/IL and nanohybrid indicate three and two weight loss steps, respectively. The first weight loss step occurs in the temperature range of 25–170 °C and is attributed to the evaporation of adsorbed water molecules (15% for mTEG-CS-Schiff-base/IL and 1.5% for nanohybrid). In the second stage, a total weight loss of 50% for mTEG-CS-Schiff-base/IL and 4.8% for the nanohybrid is observed. This weight loss is associated with the decomposition of the Schiff-base units and the chitosan chains as a polysaccharide.

**Figure 8:**
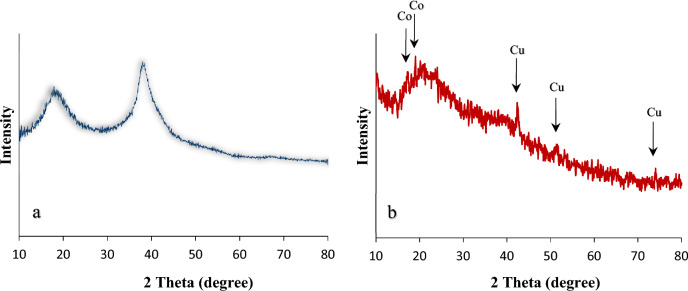
XRD patterns of mTEG-CS-Schiff-base/IL (**a**) and mTEG-CS-Co/Cu-Schiff-base/IL nanohybrid (**b**).

As illustrated in Fig. 8, XRD patterns for mTEG-CS-Schiff-base/IL and nanohybrid are displayed. The chitosan monomer structure contains strong intermolecular hydrogen bonds. In the XRD pattern of mTEG-CS-Schiff-base/IL, two characteristic peaks in the range of 2θ = 10°-20° indicate its semi-crystalline nature (Fig. 8a). However, the XRD pattern of the nanohybrid (Fig. 8b) displays a range of peaks varying from small to intense. The broa peak observed at 10°-20° in the XRD pattern of the mTEG-CS-Co/Cu-Schiff-base/IL nanohybrid corresponds to the amorphous nature of chitosan. Additionally, there are diffraction peaks attributed to cobalt (two weak sharp peaks) and copper (three weak to moderate peaks) observed at 17.20°, 19.05° and 42.35°, 51.70°, 74.05°, respectively^96, 97^. These peaks indicate the crystalline changes resulting from the coordination of metals with the modified chitosan polymer. Moreover, the deformation of hydrogen bonds during condensation leads to a decrease in the crystallinity of chitosan.

**Figure 9:**
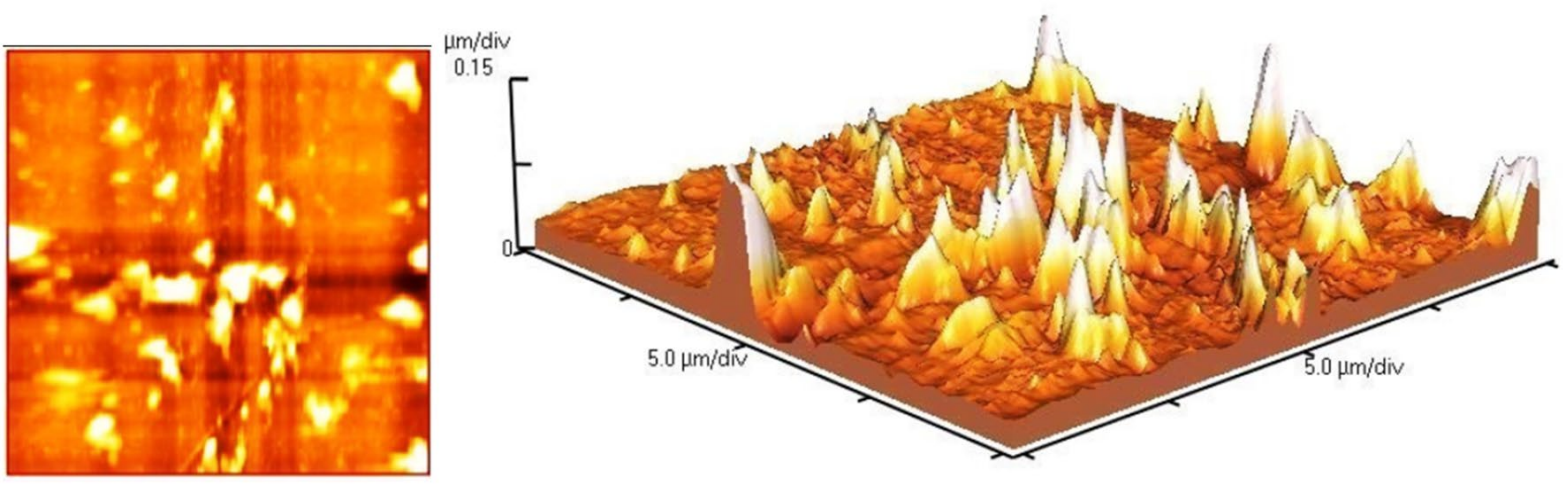
AFM images of mTEG-CS-Co/Cu-Schiff-base/IL nanohybrid.

Real-time visualization of the nanohybrid is possible through in situ atomic force microscopy (AFM). Figure 9 illustrates the topographical structure of thin films from AFM images. Different parameters, such as root mean square roughness (RMS), can be utilized to quantify the topographical characteristics of a surface. The RMS roughness of an mTEG-CS-Co/Cu-Schiff-base/IL nanohybrid thin film was measured to be 26.64 nm. In the 3D image, multiple peaks representing the porous and uneven surface of the nanohybrid are evident. This suggests that the modification of the chitosan surface leads to an increase in number of pores and surface roughness.

### The catalytic activity of the mTEG-CS-Co/Cu-Schiff-base/IL nanohybrid in the synthesis of chromeno pyrimidine via* multicomponent TOP*

**Figure 10:**
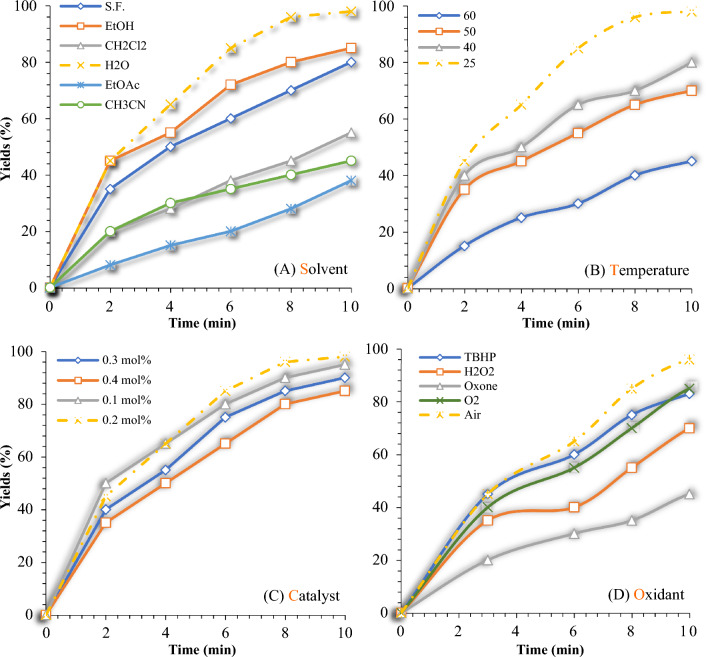
The screening effect of the solvent (**a**), temperature (**b**), amount catalyst (**c**), and oxidant (**d**) on the oxidation of alcohols reaction. Oxidation of alcohols reaction conditions: Benzyl alcohol (1 mmol), mTEG-CS-Co/Cu-Schiff-base/IL (0.2 mol%), H_2_O, R.T, open air.

After successfully synthesizing and identifying mTEG-CS-Co/Cu-Schiff-base/IL nanohybrid, its catalytic performance was probed in the chromenopyrimidine-2,5-dione reaction. The optimization of reaction parameters was studied by the multi component reaction between benzyl alcohol, 4-hydroxycumarine, and urea as a model reaction. First, the model reaction was carried out in the absence of nanohybrid and in the presence of water as a solvent under reflux conditions. Without the nanohybrid, only trace amounts of the desired product were observed, demonstrating the importance of the catalyst in promoting the reaction. However, before investigating the chromenopyrimidine-2,5-dione reaction, benzyl alcohol was used as a model compound to investigate the oxidation activity of the catalyst. The influence of solvent, catalyst amount, diverse oxidants, and temperature on the oxidation of benzyl alcohol to benzaldehyde was investigated (Fig. 10). First, as can be seen in Fig. 10a, a range of solvents including water, dichloromethane, ethanol, ethyl acetate, acetonitrile and a solvent-free reaction was investigated. Among the solvents investigated, water emerged as the preferred solvent for this reaction. Through experimentation and data analysis, it was observed that ethyl acetate, dichloromethane, and acetonitrile exhibited lower improvement in efficiency compared to water, in the oxidation reaction of alcohol. The low efficiency of solvents in this reaction may be attributed to their lower solubility, unintended interferences, and unfavorable physical and chemical properties. Next, the effect of reaction temperature was examined in the oxidation reaction of alcohol (Fig. 10b). The temperature range investigated was between 25 to 60 °C, and it was found that a temperature of room temperature (25 °C) yielded better results compared to higher temperatures. One reason for the decrease in efficiency at higher temperatures can be attributed to the increase in reaction rate. As the temperature rises, the reaction rate also increases. This can lead to the formation of byproducts or impurities that can decrease the overall efficiency. Therefore, to improve efficiency, the optimal temperature for this reaction could be room temperature. In the following, the oxidation reaction of alcohol (Fig. 10c) was investigated using varying amounts of mTEG-CS-Co/Cu-Schiff-base/IL nanohybrid. Among the different catalyst concentrations tested, it was found that 0.2 mol% of the catalyst yielded the best results. Further increasing or decreasing the catalyst concentration did not significantly improve the yield. Higher catalyst concentrations may saturate the reaction system, leading to overcrowding of active sites and reduced efficiency. Conversely, lower catalyst concentrations may not provide enough active sites for effective catalysis, resulting in lower yields. Therefore, 0.2 mol% of the catalyst appears to strike the right balance, maximizing the yield in the oxidation reaction of alcohol. Finally, the role of oxidants in oxidation reactions is crucial, and in order to identify the most effective oxidant, different strong and weak oxidants were evaluated under the same reaction conditions (Fig. 10d). Among the oxidants tested (O_2_, H_2_O_2_, Air, TBHP, and Oxone), aerobic conditions played a significant role in conversion and selectivity. This suggests that atmospheric oxygen present in the air serves as an efficient oxidizing agent for the oxidation reaction of interest. The preference for air as the oxidant could be attributed to its availability, cost-effectiveness, and compatibility with the reaction system. The use of other oxidants, either strong or weak, did not yield as favorable results. The presence of a strong oxidant in the alcohol oxidation reaction led to a decrease in efficiency. The strong oxidant can compete with the catalyst and extract active species from it, resulting in reduced catalyst activity and overall reaction efficiency. Also, the strong oxidant can generate undesired side products, which can negatively impact the overall yield. Furthermore, it can interfere with electron transfer processes by absorbing electrons from the catalyst, disrupting the electron transfer pathway and leading to decreased reaction efficiency. Lastly, the presence of a strong oxidant can cause damage and alteration to the catalyst, further diminishing its catalytic activity and reducing the overall reaction efficiency.

Under optimal reaction conditions, a variety of primary benzylic alcohols were oxidized to assess the applicability of the protocol (R.T in H_2_O under aerobic conditions). Electron-withdrawing and higher steric hindrance increased reaction times for benzyl alcohols (Table 1, entries 2, 4, 6, 7, 9). The results showed no significant difference in the efficiency of alcohols bearing electron-withdrawing and electron-donating groups, with excellent efficiency for all derivatives, without excessive oxidation to carboxylic acid or ester.

**Table 1:** Oxidation of benzyl alcohols to corresponding aldehydes in the present of mTEG-CS-Co/Cu-Schiff-base/IL nanohybrid.


Entry	Substrate (R)	Product	Time (min)	Yield^a^ (%)
1	H	Benzaldehyde	10	98
2	2-Cl	2-Chlorobenzaldehyde	10	93
3	4-Cl	4-Chlorobenzaldehyde	10	90
4	2-Br	2-Bromobenzaldehyde	15	87
5	4-Br	4-Bromobenzaldehyde	25	83
6	2-NO_2_	2-Nitrobenzaldehyde	30	87
7	4-NO_2_	4-Nitrobenzaldehyde	30	90
8	4-MeO	2-Methoxybenzaldehyde	20	95
9	2-OH	2-Hydroxybenzaldehyde	20	85
10	4-CH_2_OH	Terephthalaldehyde	45	80

Reaction conditions: benzyl alcohol derivatives (1 mmol), mTEG-CS-Co/Cu-Schiff-base/IL (0.2 mol%), H_2_O, R.T, open Air.

^a^Isolated product yields.

Following the successful oxidation of alcohols, we investigated the effectiveness of the catalyst mTEG-CS-Co/Cu-Schiff-base/IL in the synthesis of chromenopyrimidine via a multicomponent tandem oxidation process. The chromenopyrimidine derivatives were synthesized using the nanohybrid under optimized conditions, involving the reaction of alcohols or bis-aldehydes with 4-hydroxycoumarin and urea, resulting in yields of 85–98% (as shown in Table 2). It can be inferred from Table 2 that the product yields of benzyl alcohols with electron-withdrawing groups are higher compared to those with electron-donating groups. Importantly, the reactions proceeded smoothly without generating any side products, particularly oxidation products. These observations highlight the remarkable catalytic activity and selectivity of the catalyst for synthesis of chromenopyrimidines from alcohols and bis-aldehydes with 4-hydroxycoumarin and urea. Moreover, the application of this nanohybrid was evaluated for the synthesis of bis-aldehyde derivatives (as shown in Table 2), resulting in good to high yields.

**Table 2:** One-pot reaction of benzyl alcohols or bis-aldehydes with 4-hydroxycoumarin and urea catalyzed by mTEG-CS-Co/Cu-Schiff-base/IL.


Entry	Benzyl alcohol Bis-aldehyde	Product	Time (min)	Yield^a^ (%)	M.P. (°C)
1a			15	98	210–212
2b			15	95	201–203
3c			15	95	259–261
4d			15	92	205–208
5e			10	97	205–207
6f.			15	95	New 205–207
7 g			15	95	New 248–250
8 h			10	95	New 244–245
9i			10	95	New 263–265
10j			20	96	New 225–228
11 k			15	95	New 238–240
12 l			15	92	New 223–225
13 m			20	88	New 222–225
14n			15	90	New 236–238
15o			20	85	New 230–233
16p			20	95	New 244–246
17q			20	93	New 230–232
18r			20	85	New 235–237
19 s			20	90	New 242–244

Reaction conditions: Benzyl alcohols or bis-aldehydes (1.2 mmol), 4-hydroxycoumarin (1 or 2 mmol), urea (1 or 2 mmol), mTEG-CS-Co/Cu-Schiff-base/IL (0.2 mol%), H_2_O, 50 °C, aerobic condition.

^a^Isolated product yields.

To further understand the individual contributions of the components in the nanohybrid for the tandem aerobic oxidative reactions, separate control experiments were conducted for the model one-pot chromenopyrimidine reaction under the same conditions. The repeatability of the reactions, assessed by averaging the results of 4 experiments performed under identical conditions (Table 3). As shown in Table 3, the bare chitosan showed no efficiency (Table 3, entry 1). CS-Schiff-base/IL and mTEG-CS-Schiff-base/IL showed limited oxidation activity under open-air conditions, resulting in only trace amount of chromenopyrimidine was synthesized with them after 2 h (Table 3, entries 2, 3). Among the nanohybrid components, the performance of mTEG-CS-Co-Schiff-base/IL and mTEG-CS-Cu-Schiff-base/IL was valuable and significant due to the presence of copper and cobalt metals (Table 3, entries 4, 5). According to the ICP analysis, copper was found to be loaded in higher amounts than cobalt. Therefore, the mTEG-CS-Cu-Schiff-base/IL nanohybrid exhibited higher efficiency in alcohol oxidation and chromenopyrimidine (Table 3, entry 5). Interestingly, the most remarkable results were obtained using the bimetallic system mTEG-CS-Co/Cu-Schiff-base/IL as a catalyst, demonstrating its high performance in the oxidation step and confirming the synergistic effect of cobalt and copper centers in the nanohybrid for the green one-pot reaction. The obtained results clearly showed the synergistic effect and cooperative activity between the two metals, cobalt and copper, in the nanohybrid for the tandem oxidation-condensation reaction, achieved within 15 min. The catalyst metals play a crucial role in facilitating the reaction simultaneously. This study highlights that the simultaneous utilization of copper and cobalt catalyst metals in the chromenopyrimidine reaction enhances the catalyst's performance and increases the reaction efficiency, surpassing the performance achievable with each metal alone.

**Table 3:** Control experiments designed for alcohol oxidation and preparation of chromeno pyrimidine reaction in one pot.

Entry	Catalyst	Amount of metal loading per gram (%W)	Oxidation of alcohol^a^ temp./time/yield (%)	Chromenopyrimidine^b^ temp./time/yield (%)
1	Chitosan	–	70 °C/6 h/–	80 °C/45 min/–
2	CS-Schiff-base/IL	–	70 °C/2 h/Trace	80 °C/45 min/Trace
3	mTEG-CS-Schiff-base/IL	–	70 °C/2 h/Trace	80 °C/45 min/Trace
4	mTEG-CS-Co-Schiff-base/IL	7.4	60 °C/60 min/90	80 °C/90 min/82
5	mTEG-CS-Cu-Schiff-base/IL	7.9	60 °C/45 min/95	80 °C/90 min/90
6	mTEG-CS-Co/Cu-Schiff-base/IL	6.8 (Cu) 6.3 (Co)	R.T/10 min/98	50 °C/15 min/98

^a^Reaction conditions: Benzyl alcohol derivatives (1 mmol), nanohybrid (0.2 mol%), H_2_O, open Air.

^b^Reaction conditions: Benzyl alcohol (1.2 mmol), 4-hydroxycoumarin (1 mmol), urea (1 mmol), nanohybrid (0.2 mol%), H_2_O, aerobic condition.

**Figure 11:**
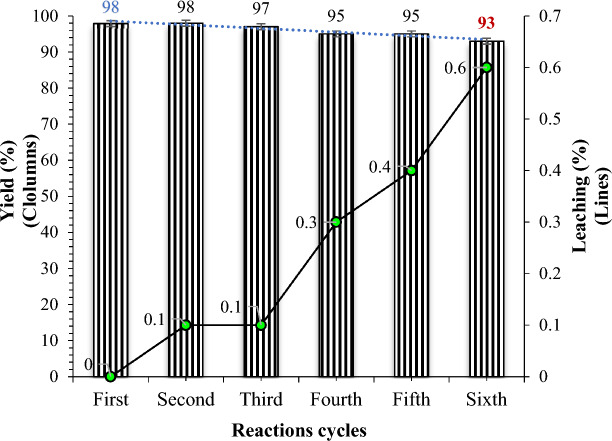
The reusability of catalysts for synthesis of chromeno pyrimidine.

To check the reusability of the nanohybrid, a recycling test was performed for the model reaction (benzyl alcohol, urea, and 4-hydroxycoumarin) under optimized conditions. The presence of the hydrophilic mTEG and imidazole tag in the catalyst facilitated the movement of organic molecules toward the catalyst, promoting faster interactions and reactions. However, the mTEG-CS-Co/Cu-Schiff-base/IL catalyst itself was hydrophobic and remained dispersed in the aqueous phase without any affinity for the organic solution (Fig. 11). Consequently, the catalyst remained in the aqueous phase until the product was extracted using EtOAc. Then, the aqueous phase was recharged with benzyl alcohol, urea, and 4-hydroxycoumarin for the subsequent run without separating the catalyst. As shown in Fig. 11, a slight decrease in catalytic activity of the nanohybrid was observed after the 6th run, with a decrease of only 5%. Furthermore, the amount of copper and cobalt leaching into the solution during the chromenopyrimidine reaction after the 6th run was measured using ICP analysis of the nanohybrid. The analysis revealed that the weight percentages of copper (Cu) and cobalt (Co) remained unchanged from their corresponding fresh values: Cu 6.5 w%, Co 6.1 w%. These findings indicate that the nanohybrid catalyst exhibited very low leaching rates, which is environmentally significant. On the other hand, to assess the durability and structure of the catalyst, the recovered catalyst was subjected to FT-IR analyze after the 6th run. Figure 1s displays the FT-IR analysis results for the recovered catalyst, confirming that the chemical structure of the catalyst remained intact throughout the recycling process (Fig. 1S: See spectral data in SI File).

**Figure 12:**
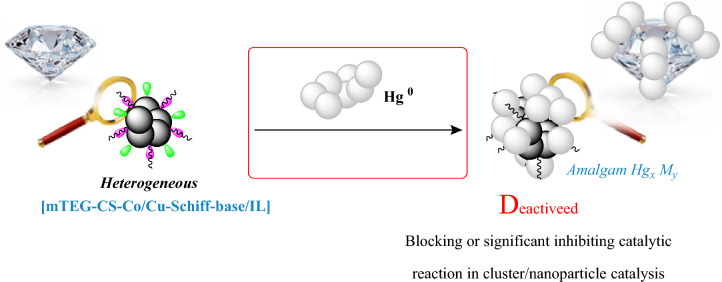
Operational origin of Hg testing in mechanistic studies of catalytic systems.

Finally, to indicate the heterogeneous nature of the catalyst, Hg poisoning tests and hot filtration were conducted on the chromeno pyrimidine synthesis under optimal conditions (Fig. 11). In this method, metallic mercury is used to poison M^0^ clusters or nanoparticles that function as catalytic centers, while it remains inert towards molecular metal complexes. If the addition of metallic mercury inhibits the catalytic reaction, it suggests the involvement of a cluster or nanoparticle in the catalytic mechanism (Fig. 12).

**Figure 13:**
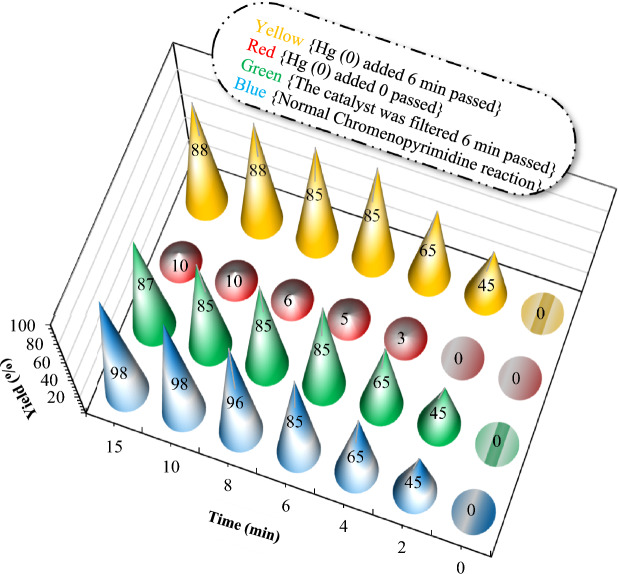
Results of mercury poisoning and hot filtration experiments on the one-pot reaction of chromenopyrimidine of benzyl alcohol with 4-hydroxycoumarin, and urea.

In Fig. 13, the blue cone represents progress of the model reaction in the presence of the mTEG-CS-Co/Cu-Schiff-base/IL as the catalyst in the range of 0–15 min. In the green cone experiment, the catalyst was removed from the reaction mixture through filtration after six minutes, and the reaction was allowed to continue, but no significant progress was observed. In the two separate experiments denoted by the red and yellow cone, the red cone represents the addition of mercury (Hg) from the beginning of the reaction. In this case, the reaction only progressed to 10% completion after 15 min, indicating that the presence of mercury inhibited the catalytic activity of the mTEG-CS-Co/Cu-Schiff-base/IL catalyst. On the other hand, the yellow cone represents the addition of mercury to the reaction vessel after six minutes. In this scenario, the reaction did not progress significantly, suggesting that the addition of mercury at this point did not significantly affect the already initiated catalytic process. These observations indicate that the presence of mercury has a negative impact on the catalytic activity of the mTEG-CS-Co/Cu-Schiff-base/IL catalyst.

Table 4 presents a comparison of the nanohybrid catalyst with various other catalysts previously employed in the same reaction. The results demonstrate that our designed catalyst exhibits efficient catalytic activity compared to the other catalysts tested. The reaction proceeds to completion with the appropriate amount of mTEG-CS-Co/Cu-Schiff-base/IL catalyst, and it provides the desired products from alcohol derivatives in a short period of time. It was found that these monometallic catalysts possess a certain degree of catalytic activity on their own. Furthermore, the catalytic performance of mTEG-CS-Co-Schiff-base/IL and mTEG-CS-Cu-Schiff-base/IL as monometallic catalysts was also investigated. However, when comparing the catalytic activity of the nanohybrid with that of mTEG-CS-Co-Schiff-base/IL and mTEG-CS-Cu-Schiff-base/IL alone, it was observed that the bimetallic nanohybrid exhibits higher catalytic activity. This enhanced catalytic performance can be attributed to the role of cobalt and copper in dispersing the chitosan surface and the synergistic effect between metals. The presence of both cobalt and copper increases the number of active sites available for the catalytic reaction, leading to improved catalytic activity. It should be noted that the objective of this study is to introduce a catalyst with potential applications in enhancing organic reactions. The synthesis of chromenopyrimidine-2,5-dione derivatives serves as a specific model for organic transformation in this context.

**Table 4:** The comparison of the nanohybrid activity of mTEG-CS-Co/Cu-Schiff-base/IL with previously reported catalysts.

Entry	Catalyst	Time (min)	Reaction condition	Yield (%)	References
1	H_5_BW_12_O_40_	30	Reflux, water	97	^30^
2	HPA@Methenamine-HNTs	20	Reflux, water	97	^31^
3	CH_3_SO_3_H	30	Ultrasound	80	^98^
4	HSO_3_Cl	5	Ultrasound	96	^98^
5	CH_3_C_6_H_4_SO_3_H	60	Ultrasound	75	^98^
6	HPA@HNTs-C	4	Ultrasound	95	^99^
7	Ag@CDNS-SBA-15	25	Water, 55 °C, 125 W	95	^100^
8	SDS	360	Water, Reflux, Acetic acid	78	^101^
9	SLS	300	Water, RT	95	^102^
10	Copper iodide nanoparticles	50	Solvent-free, 79 °C	92	^103^
11	mTEG-CS-Co/Cu-Schiff-base/IL	15	H_2_O, 50 °C	98	This work

**Figure 14:**
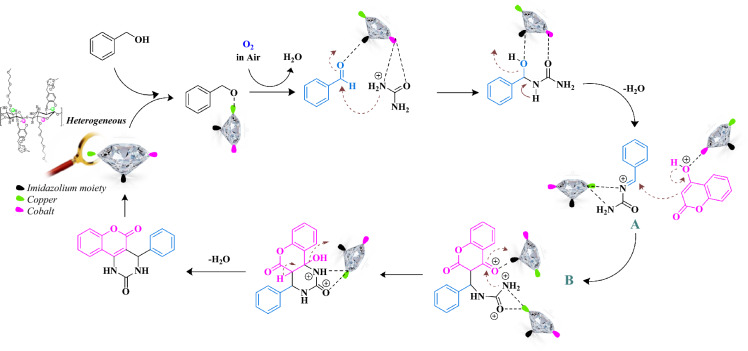
Proposed Mechanism for the Multicomponent TOP Using mTEG-CS-Co/Cu-Schiff-base/IL nanohybrid as a multifunctional catalyst.

Figure 14 proposes a mechanism for the reaction of chromenopyrimidine synthesis using the newly developed hydrophilic heterogeneous cobalt/copper bimetallic catalyst. The mechanism involves the synergistic effect of both cobalt and copper metals in the catalyst to enhance the efficiency of the reaction. The proposed mechanism suggests that both copper and cobalt metals play a role in the oxidation of alcohol to aldehyde. Copper metal, which is present in a higher amount based on ICP analysis, acts as the primary activator, while cobalt metal acts as the electron acceptor. In aerobic conditions, aldehydes are produced from alcohols through dehydration in the presence of copper metal. Then, the cobalt metal in the catalyst activates the urea by forming a bond with the carbonyl and amine group in the compound. This interaction prepares the reaction to proceed and enhances the reactivity of the urea. In this way, cobalt metal as an electron acceptor plays an important role in reducing the electronic potentials of the reaction and increasing the reactivity. In the following, the nanohybrid enables the formation of intermediate (A) by facilitating the Knoevenagel condensation of the aldehydes formed in situ with urea, leading to the synthesis of the imine. Then, the active sites of copper in the catalyst coordinate with the imine group (A) and undergo Michael's addition, which involves the nucleophilic addition of 4-hydroxycoumarin. This results in the formation of intermediate B. Intermediate B undergoes nucleophilic attack of amine to the carbonyl group of 4-hydroxycoumarin. This step leads to the formation of the chromenopyrimidine product after the removal of a water molecule. Finally, the nanohybrid is regenerated for the next run, completing the catalytic cycle. Overall, the proposed mechanism highlights the intricate steps involved in the chromenopyrimidine synthesis and elucidates how the bimetallic catalyst facilitates each stage, providing insight into the catalytic process enabled by the cobalt/copper nanohybrid catalyst (Fig. 14).

## Conclusion

The paper describes the development and characterization of a new hydrophilic heterogeneous cobalt/copper bimetallic catalyst for synthesizing chromopyrimidine derivatives. The catalyst composition includes chitosan-Schiff base, imidazolium moieties as ionic liquids, and mono-methoxy triethylene glycol (mTEG) as a phase transfer functional group. This catalyst enables a multicomponent tandem oxidation process starting from alcohols, offering an economically viable and environmentally sustainable alternative to conventional methods. The paper emphasizes several advantages of this process. Firstly, the catalyst is easy to handle and exhibits high reusability, contributing to cost-effectiveness and practicality. Additionally, the reaction time is significantly reduced, leading to shorter overall reaction periods. The work-up process is also simplified, and the yield of the desired chromopyrimidine derivatives is high. Notably, the use of water as a solvent promotes the eco-friendliness and sustainability of the process. The paper also provides a comprehensive characterization of the catalyst using various techniques such as XRD, FE-SEM, TEM, TGA, FT-IR, EDX-EDS, AFM, NMR, and ICP. Through this comprehensive characterization, the structure, composition, and effectiveness of the catalyst in synthesizing chromopyrimidine derivatives are clearly understood. Overall, the paper presents a promising approach to the synthesis of chromopyrimidine derivatives using a sustainable and eco-friendly process. The development of a new hydrophilic heterogeneous cobalt/copper bimetallic catalyst, along with its detailed characterization, makes a valuable contribution to the fields of medicinal and synthetic chemistry.

### Supplementary Information

Supplementary Information.

## Data Availability

All data generated or analyzed during this study are included in this published article (and its Supplementary Information files).
