# Supplementary material for: Advances in the greener synthesis of chromopyrimidine derivatives by a multicomponent tandem oxidation process
Source: Sci Rep. 2023 Nov 4;13:19104. doi: 10.1038/s41598-023-46004-3 (PMC10625593; doi:10.1038/s41598-023-46004-3)

Supporting Information

Advances in the Greener Synthesis of Chromopyrimidine Derivatives by a Multicomponent Tandem Oxidation Process

Pouya Ghamari kargar,[a] Ghodsieh Bagherzade [a]*

[a] *Department of Chemistry, Faculty of Sciences, University of Birjand, Birjand, 97175-615, Iran.*

**Corresponding author:* *Bagherzadeh@birjand.ac.ir*

Content:

Materials and methods……………………………..….………………..…...….…………………………..…. S2

FT-IR spectrum ………..……………………….…..…………………..…...….…………………………..…. S2

^{1}H-NMR spectra of the products………………………….……....………………….……………………. S3

Materials and methods

All chemicals were obtained from Sigma and Fluke suppliers and used without further purification. All the solvents were distilled and dried before use. Inductively coupled plasma atomic emission spectroscopic (ICP-AES) analysis was conducted using an OPTIMA 7300DV. Thermoanalyzer Shimadzu was used for thermogravimetric analysis (TGA) between 30 and 710 °C with a heating rate of 10 °C/min under a N_2 flow. A PerkinElmer Spectrum 2000 was used to record Fourier transform infrared (FT-IR) plots of all the developed materials in the range of 4000−400 cm^{-1} using KBr. Transmission electron microscopy TEM was used to characterize the powders. A JEOL 2100F transmission electron microscope operating at 200 kV and equipped with a field emission electron gun providing a point resolution of 0.19 nm was employed. The surface morphology, size, and shape of the catalyst particles were determined by field emission scanning electron microscopy (FE-SEM) of Tescan MIRA3. Ultrasonic generation was performed in an ultrasonic bath Bandelin electronic (frequency: 35 KHz). A Bruker D8 Advance instrument was employed to obtain powder X-ray diffraction data of designed materials in the 2θ range of 5−80° working at a scanning rate of 3° min^{-1}. The progress of the reactions and purity of the products were analyzed by TLC on silica gel PolyGram SILG/UV254 plates. The ^{1}HNMR spectra were recorded in a Brucker Advance 400 MHz spectrometers using DMSO-d_6 and solvents containing tetramethyl silane as the internal standard.

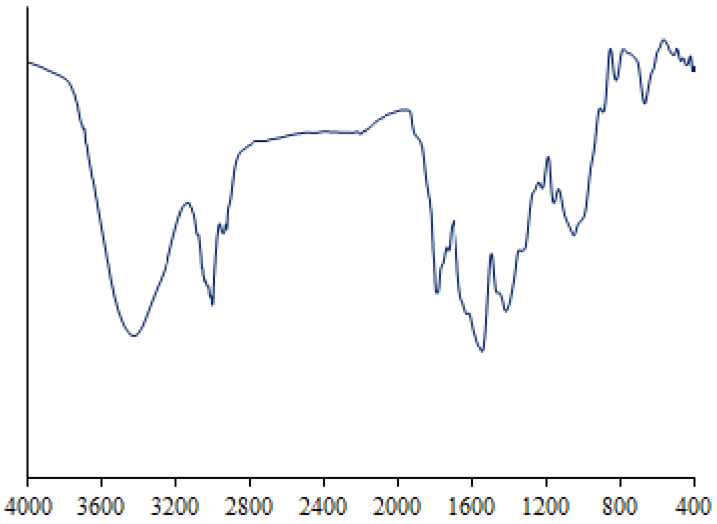

Figure 1S: FTIR spectrum of the recovered catalyst after the 6th run.

4-(2-bromophenyl)-3,4-dihydro-2H-chromeno[4,3-d] pyrimidine-2,5(1H)-dione (6f)

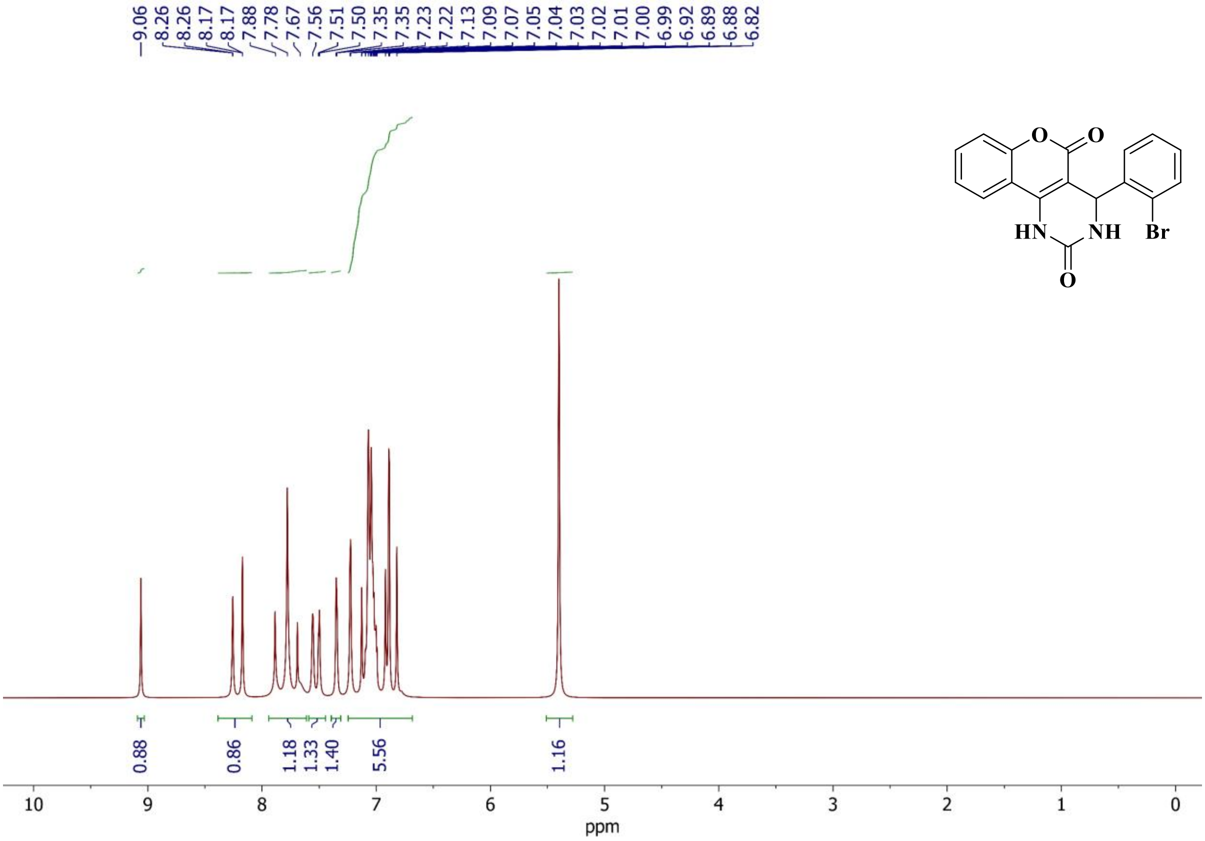

4-(4-bromophenyl)-3,4-dihydro-2H-chromeno[4,3-d] pyrimidine-2,5(1H)-dione (7g)

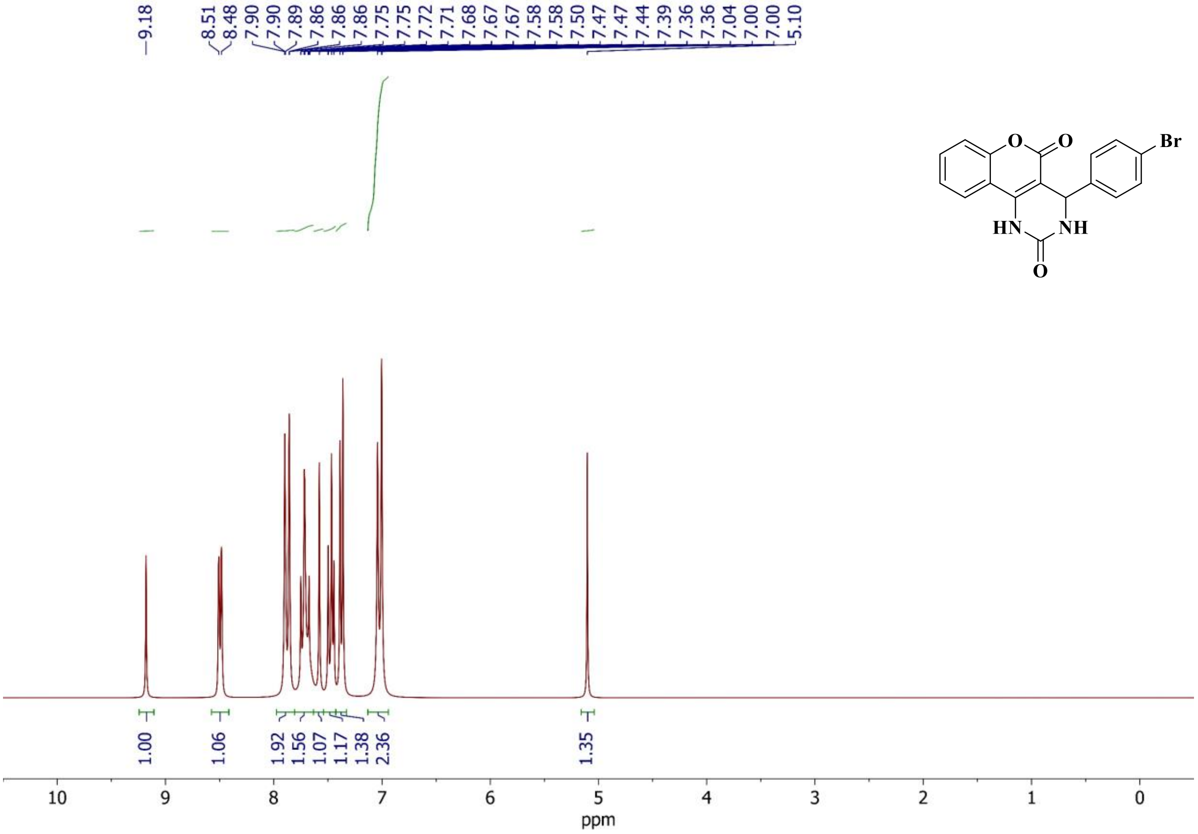

4-(2-nitrophenyl)-3,4-dihydro-2H-chromeno[4,3-d] pyrimidine-2,5(1H)-dione (8h)

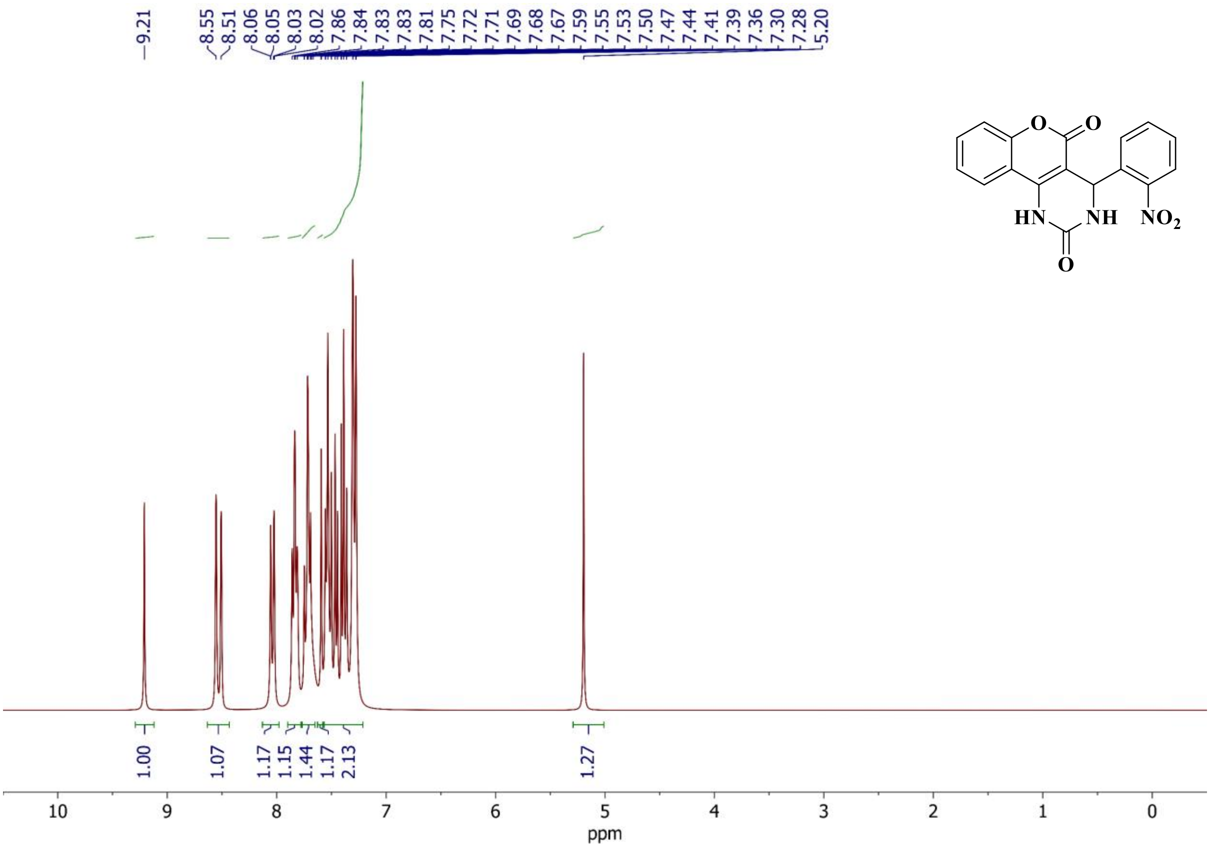

4-(4-nitrophenyl)-3,4-dihydro-2H-chromeno[4,3-d] pyrimidine-2,5(1H)-dione (9i)

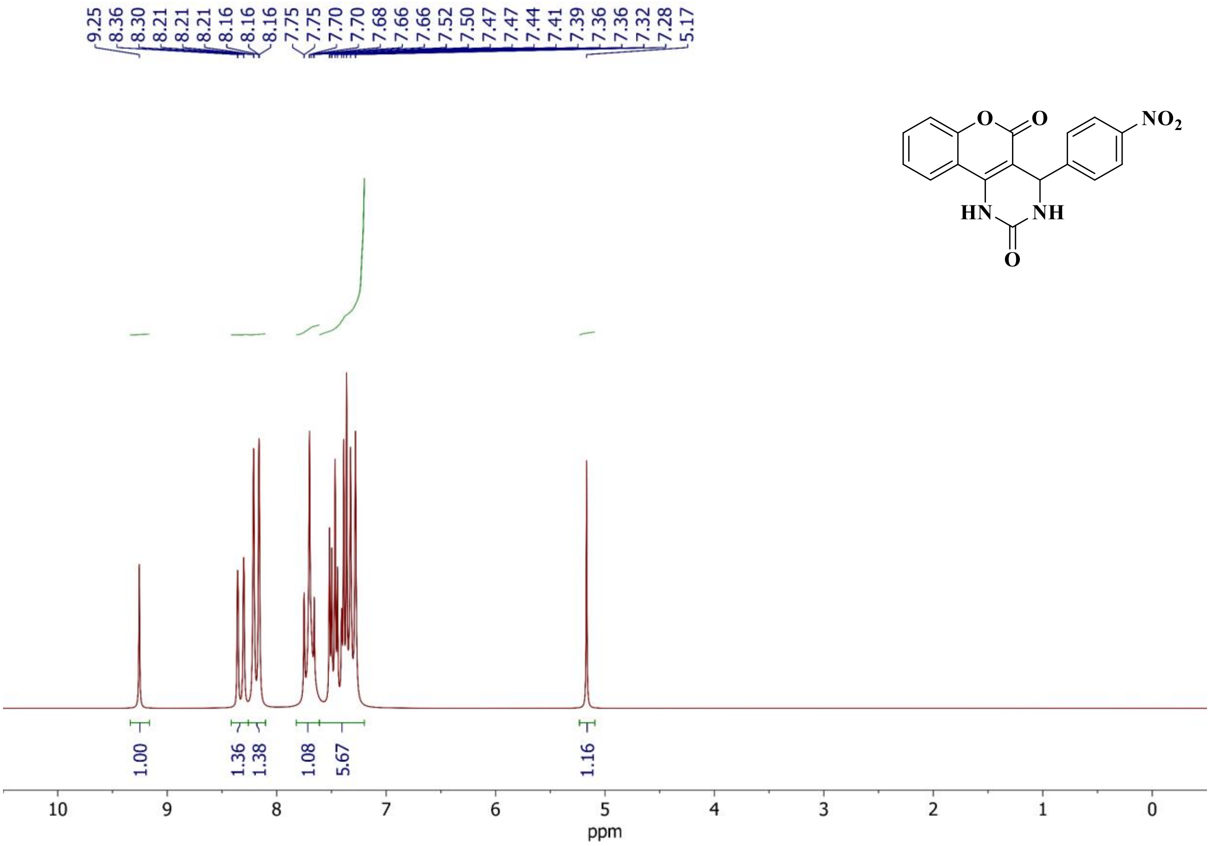

4-(2,5-dioxo-1,3,4,5-tetrahydro-2H-chromeno[4,3-d] pyrimidin-4-yl) benzaldehyde (10j)

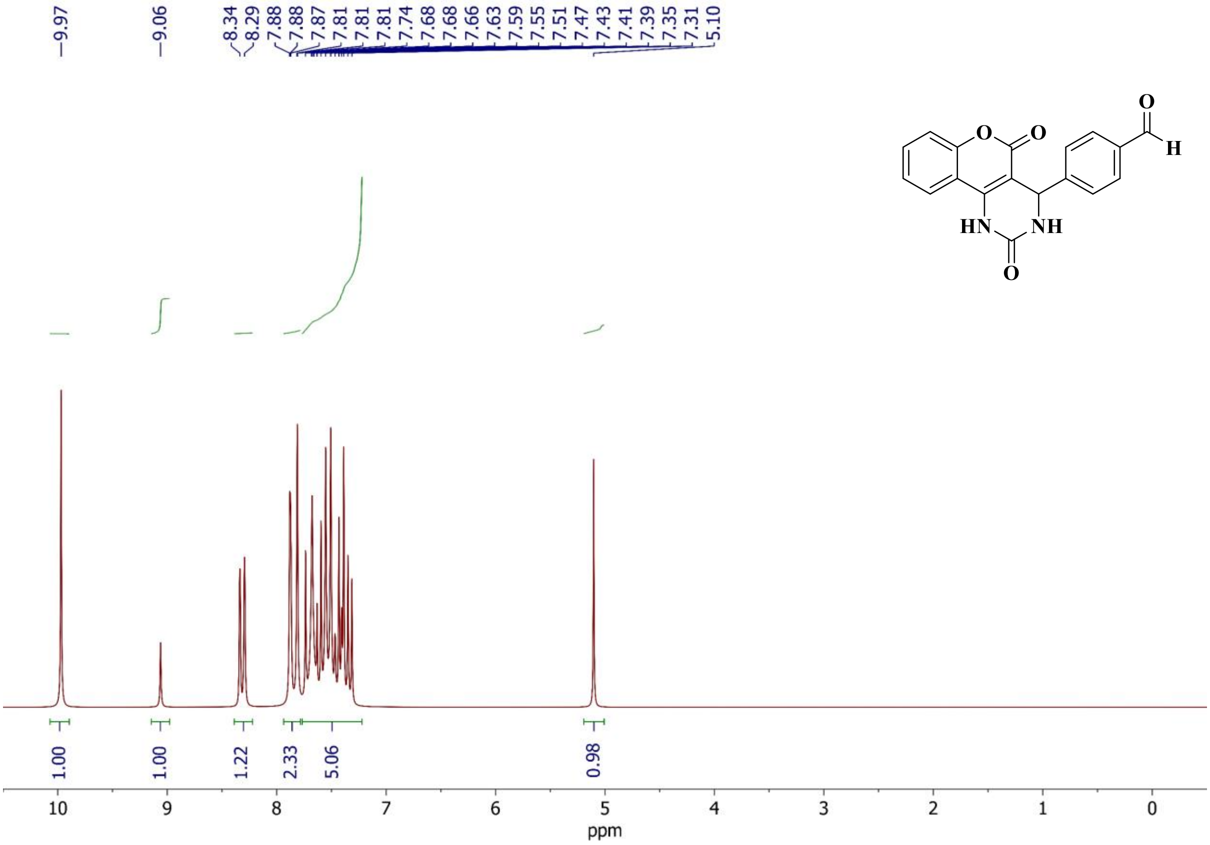

4-(4-(4-(2,5-dioxo-1,3,4,5-tetrahydro-2H-chromeno[4,3-d]pyrimidin-4-yl) phenoxy) butoxy) benzaldehyde (11k)

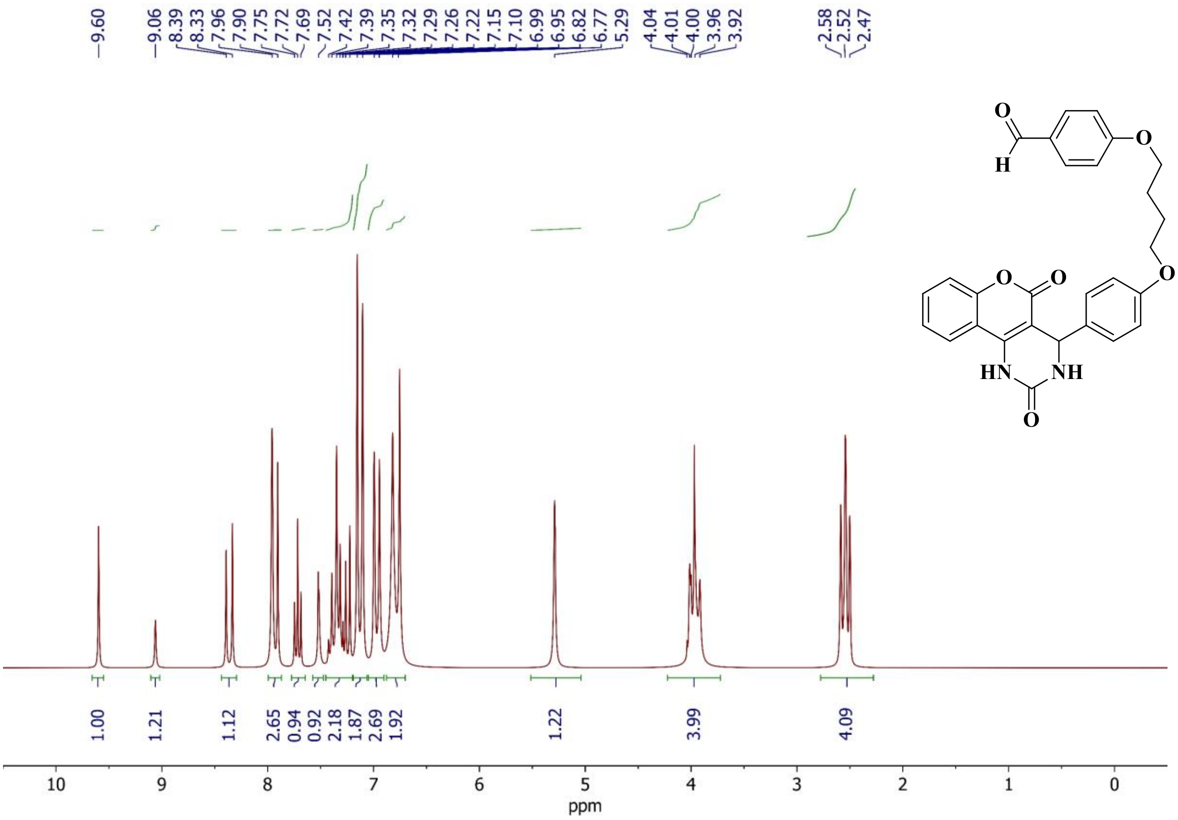

4-(2-(4-(2,5-dioxo-1,3,4,5-tetrahydro-2H-chromeno[4,3-d] pyrimidin-4-yl) phenoxy) ethoxy) benzaldehyde (12i)

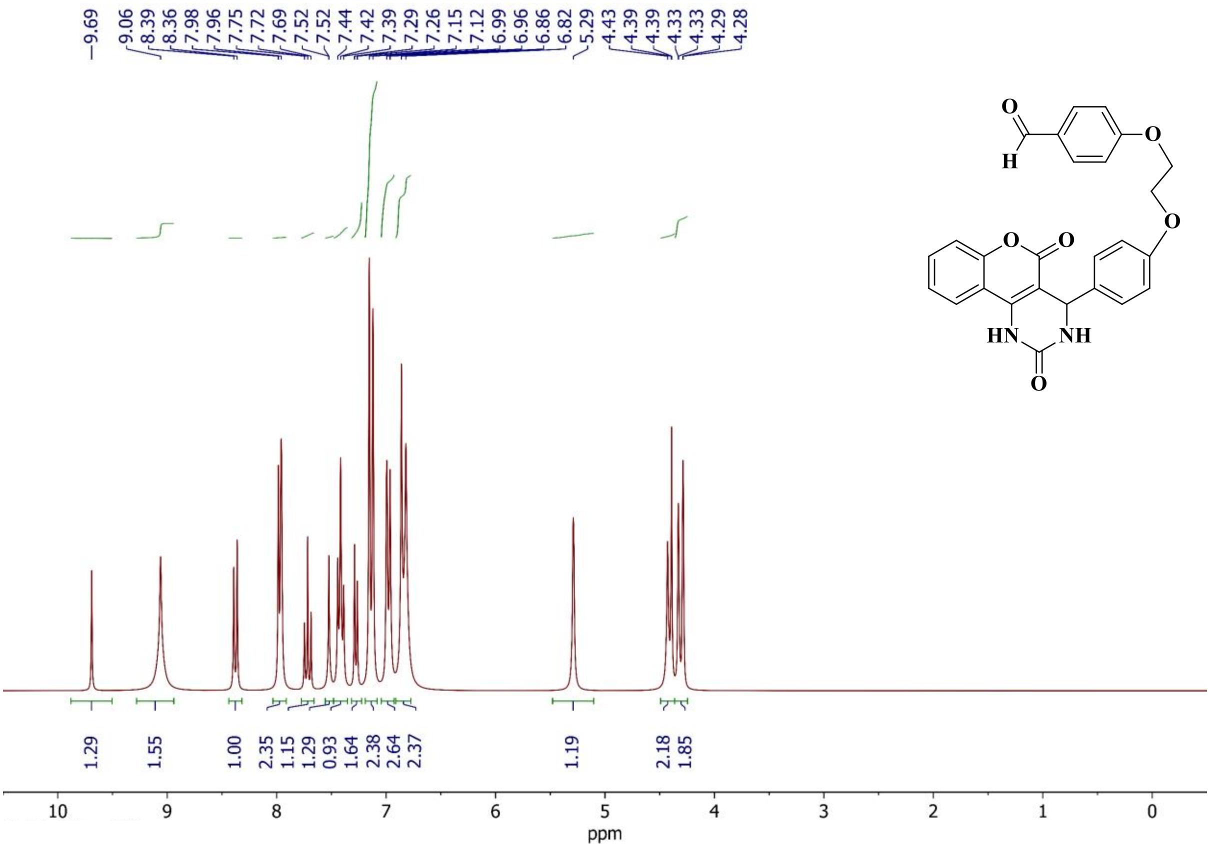

2-(4-(2-(2,5-dioxo-1,3,4,5-tetrahydro-2H-chromeno[4,3-d]pyrimidin-4-yl)phenoxy)butoxy) benzaldehyde (13m)

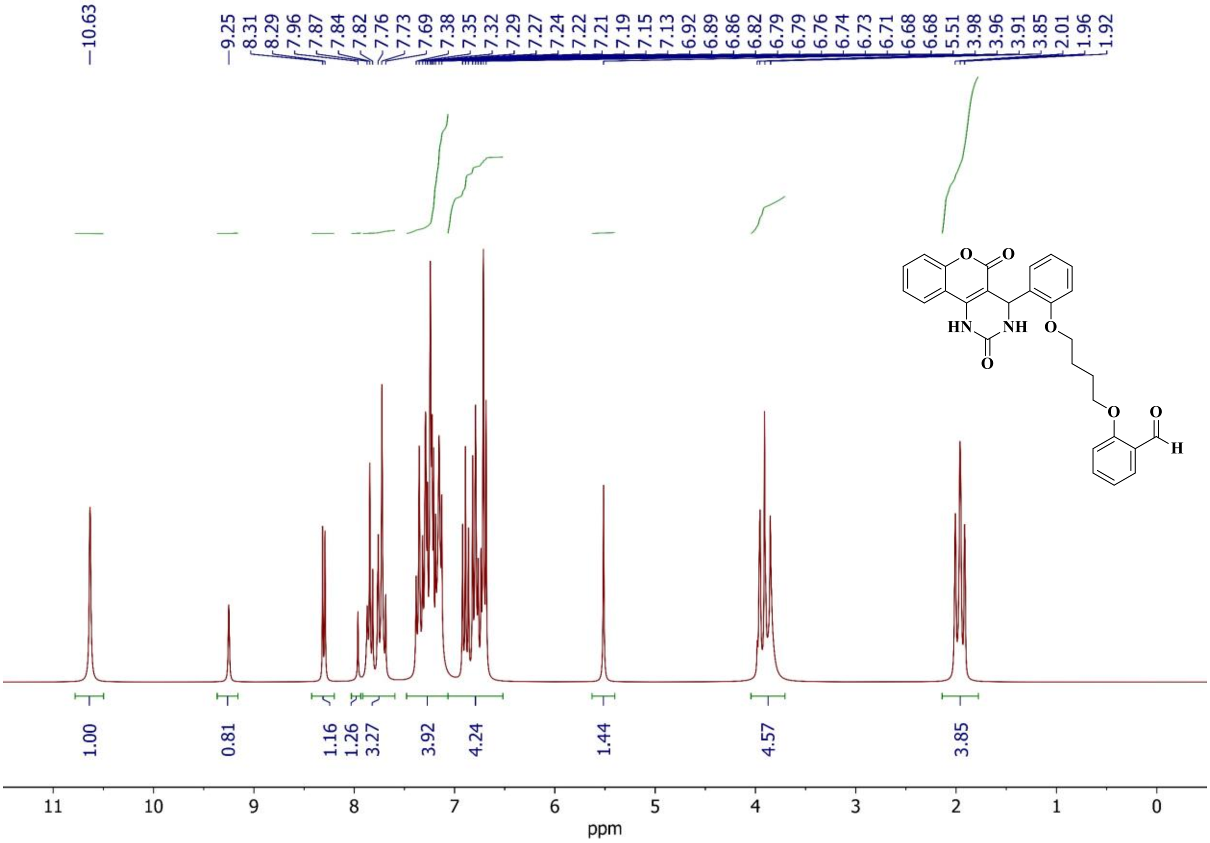

3-(2-(3-(2,5-dioxo-1,3,4,5-tetrahydro-2H-chromeno[4,3-d]pyrimidin-4-yl) phenoxy) ethoxy) benzaldehyde (14n)

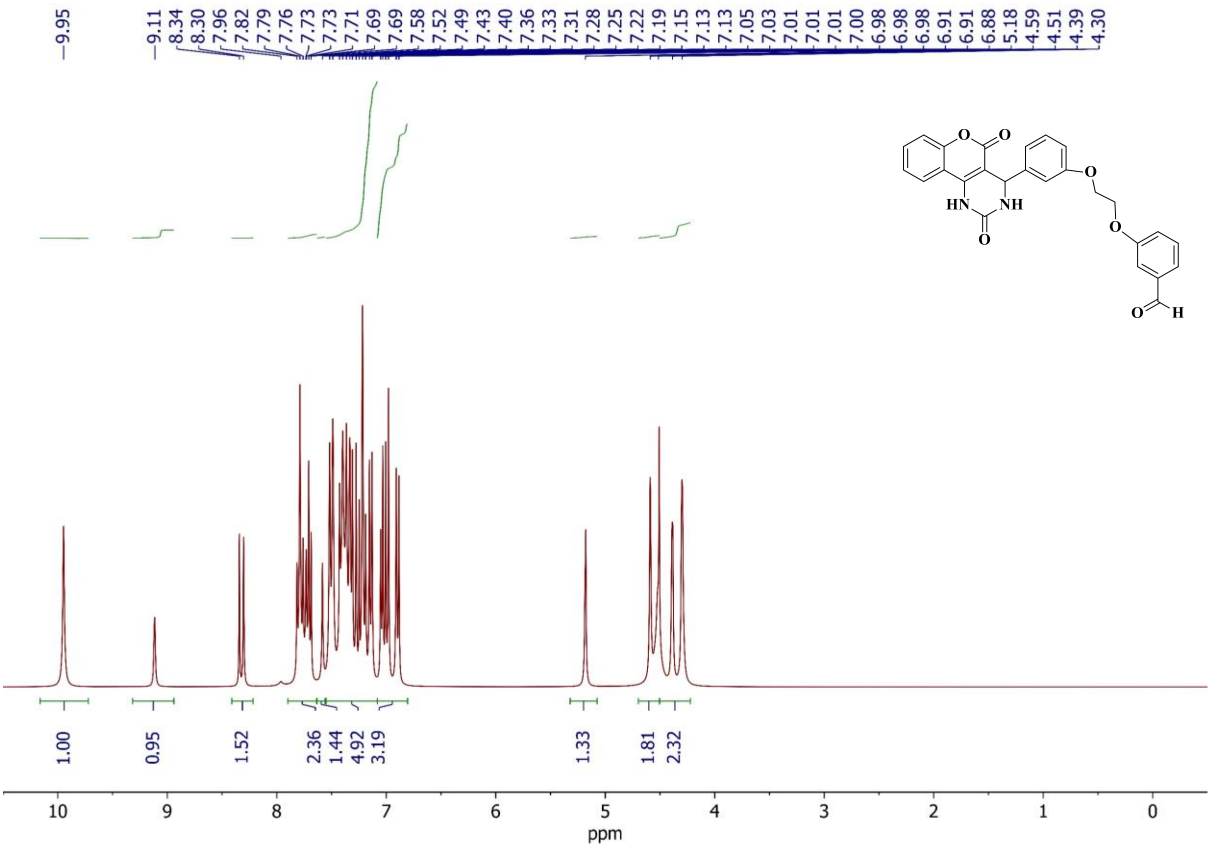

4,4'-(1,4-phenylene) bis(3,4-dihydro-2H-chromeno[4,3-d] pyrimidine-2,5(1H)-dione) (15o)

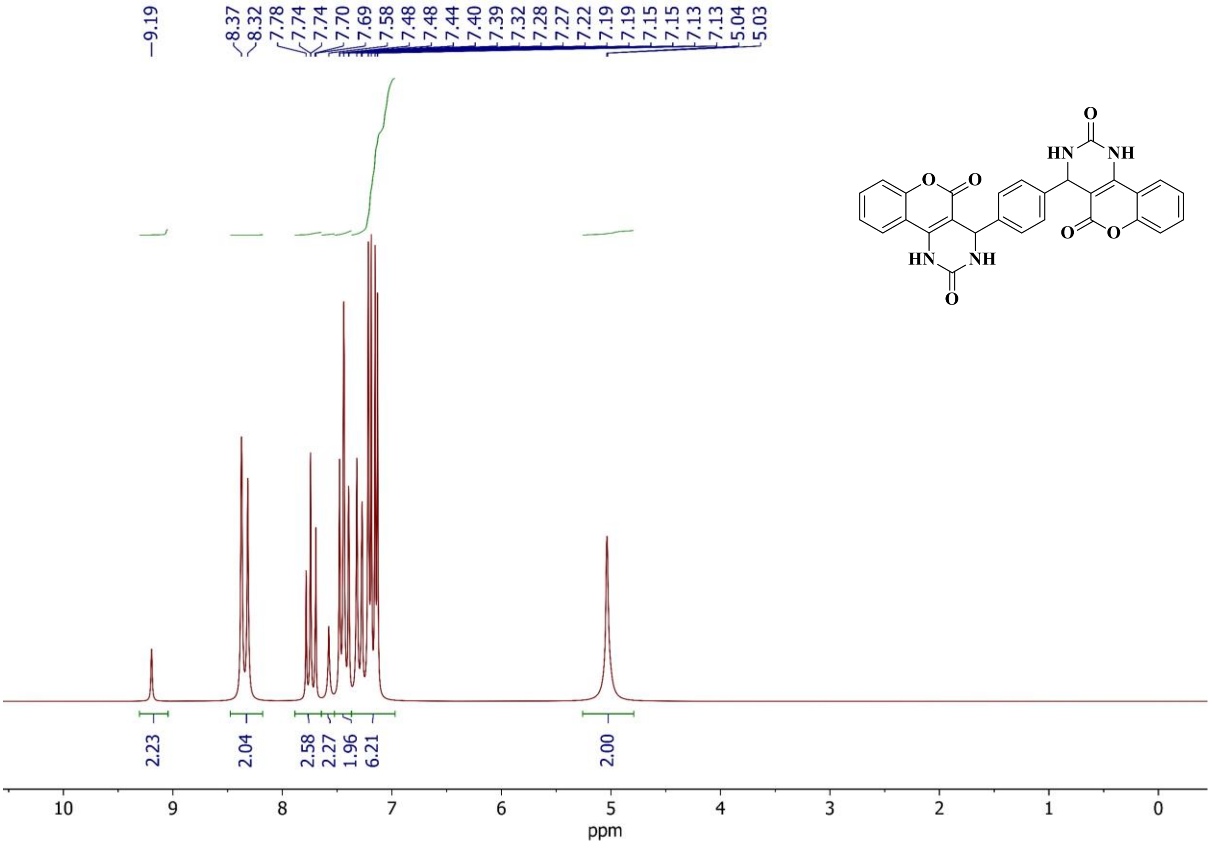

4,4'-((butane-1,4-diylbis(oxy)) bis(4,1-phenylene)) bis(3,4-dihydro-2H-chromeno[4,3-d] pyrimidine-2,5(1H)-dione) (16p)

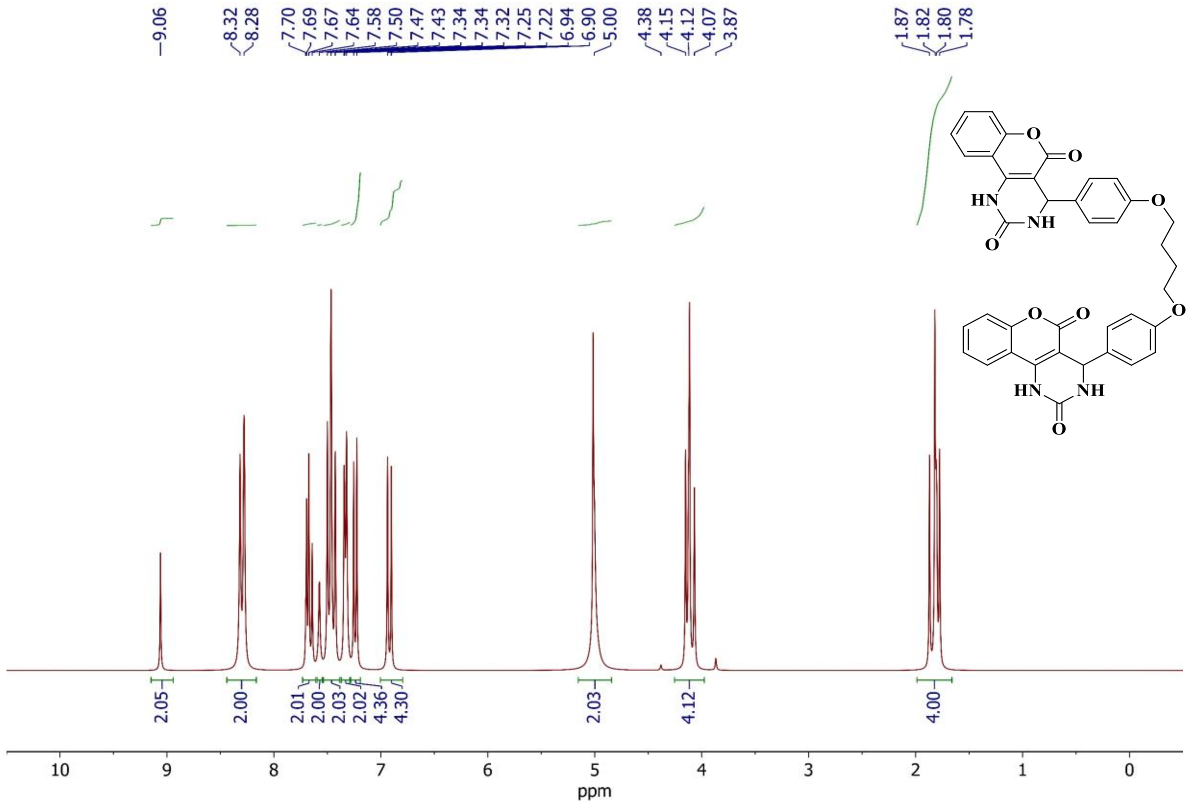

4,4'-((ethane-1,2-diylbis(oxy)) bis(4,1-phenylene)) bis(3,4-dihydro-2H-chromeno[4,3-d] pyrimidine-2,5(1H)-dione) (17q)

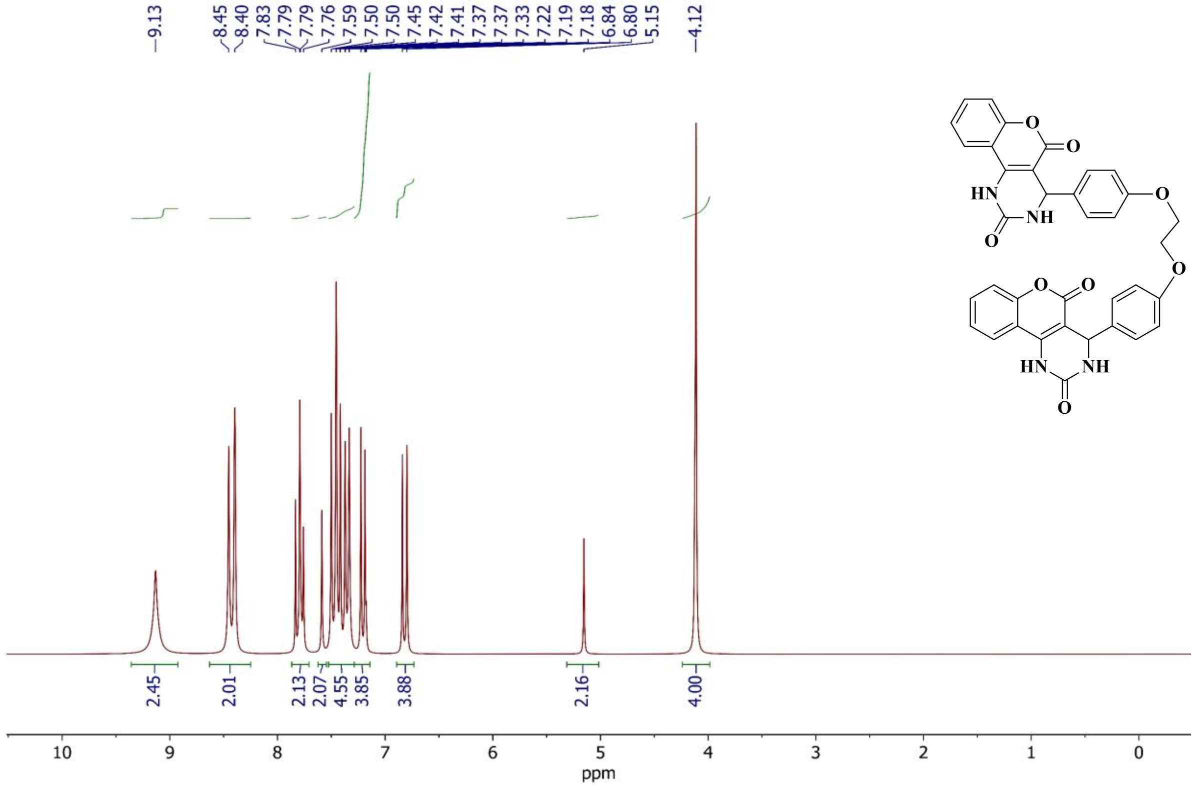

4,4'-((butane-1,4-diylbis(oxy)) bis(2,1-phenylene)) bis(3,4-dihydro-2H-chromeno[4,3-d] pyrimidine-2,5(1H)-dione) (18r)

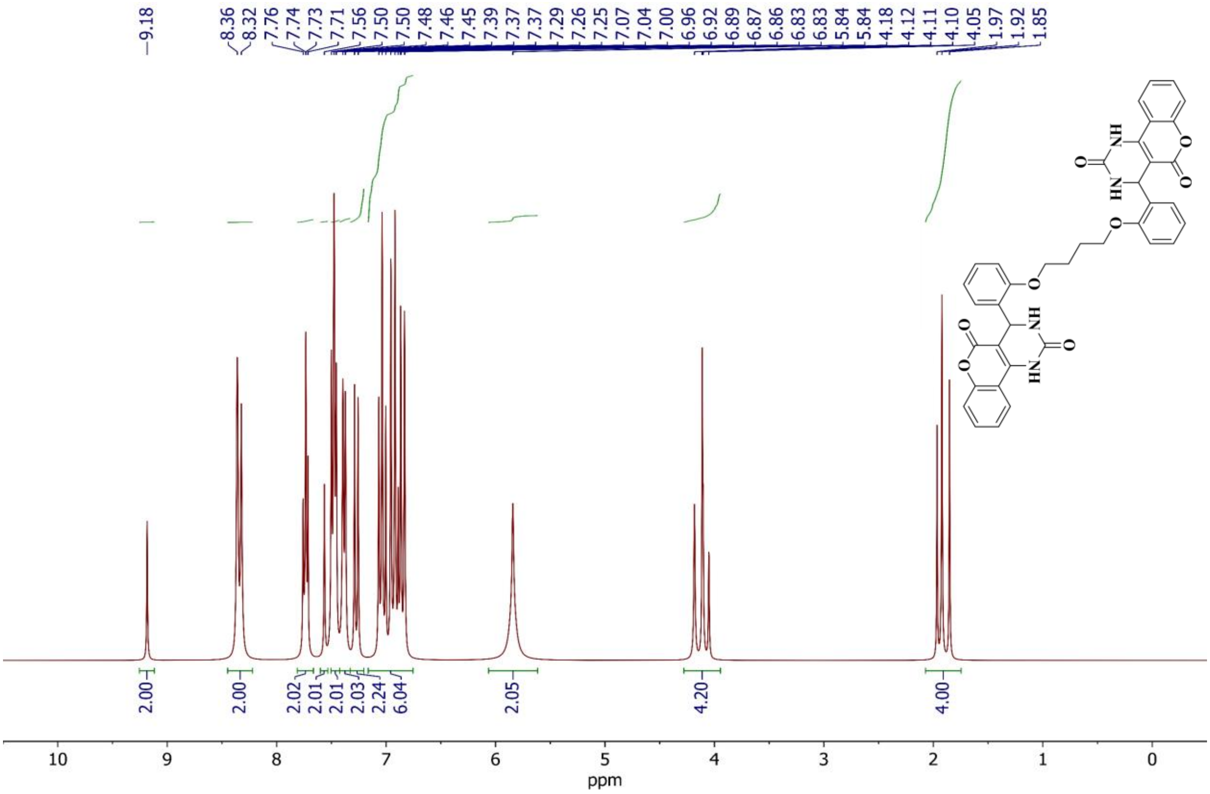

4,4'-((ethane-1,2-diylbis(oxy)) bis(3,1-phenylene)) bis(3,4-dihydro-2H-chromeno[4,3-d] pyrimidine-2,5(1H)-dione) (19s)

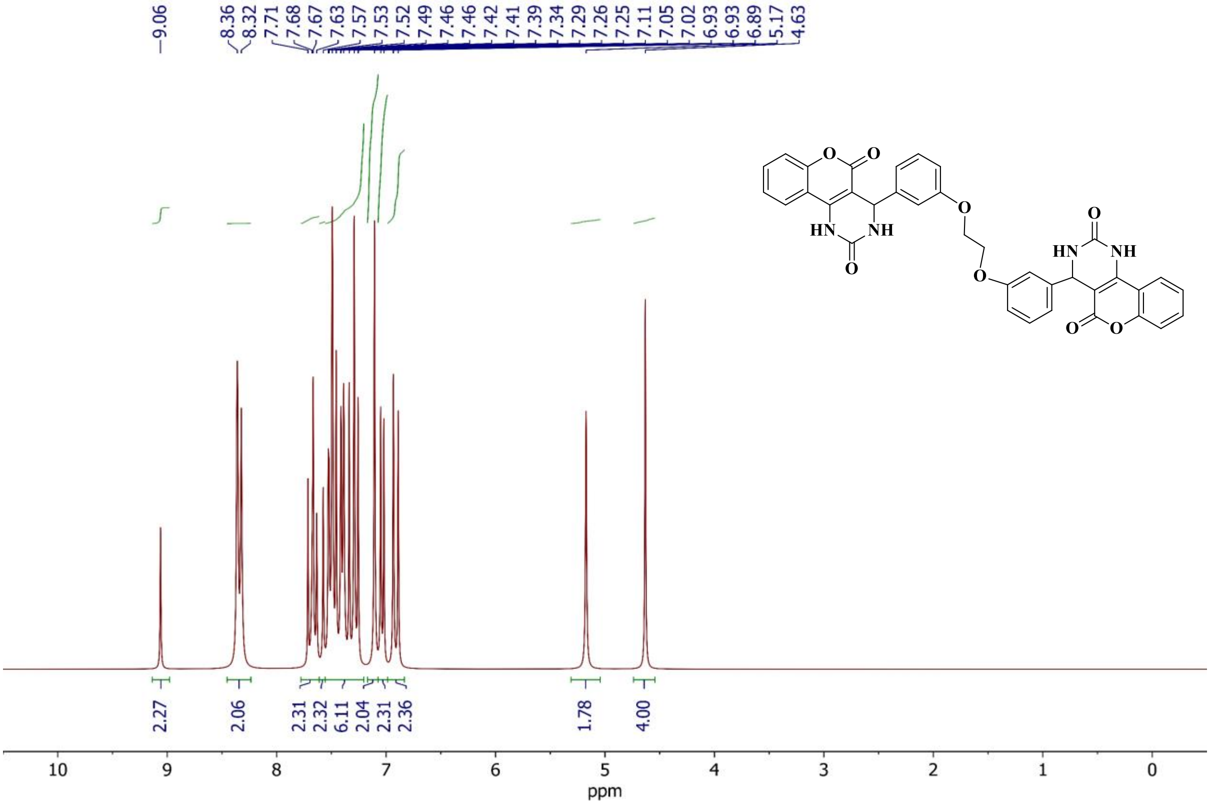

Supplement: Supplementary file 1 — Supplementary Information. [file 41598_2023_46004_MOESM1_ESM.pdf]
